# Advancements in Custom 3D-Printed Titanium Interbody Spinal Fusion Cages and Their Relevance in Personalized Spine Care

**DOI:** 10.3390/jpm14080809

**Published:** 2024-07-30

**Authors:** Kai-Uwe Lewandrowski, Shaleen Vira, John C. Elfar, Morgan P. Lorio

**Affiliations:** 1 Center for Advanced Spine Care of Southern Arizona, Division Personalized Pain Research and Education, Tucson, AZ 85712, USA; 2 Department of Orthopaedics, Fundación Universitaria Sanitas Bogotá, Bogotá 111321, Colombia; 3 Orthopedic and Sports Medicine Institute, Banner-University Tucson Campus, 755 East McDowell Road, Floor 2, Phoenix, AZ 85006, USA; shalvira@gmail.com; 4 Department of Orthopaedic Surgery, University of Arizona College of Medicine, Tucson, AZ 85721, USA; 5 Advanced Orthopedics, 499 East Central Parkway, Altamonte Springs, FL 32701, USA; mloriomd@gmail.com; 6 Orlando College of Osteopathic Medicine, Orlando, FL 34787, USA

**Keywords:** 3D printing technology, titanium interbody fusion cages, bioengineering, osteointegration, customizable implant design, patient-specific solutions, anatomical considerations, biomechanical compatibility, advanced manufacturing, clinical outcomes

## Abstract

3D-printing technology has revolutionized spinal implant manufacturing, particularly in developing personalized and custom-fit titanium interbody fusion cages. These cages are pivotal in supporting inter-vertebral stability, promoting bone growth, and restoring spinal alignment. This article reviews the latest advancements in 3D-printed titanium interbody fusion cages, emphasizing their relevance in modern personalized surgical spine care protocols applied to common clinical scenarios. Furthermore, the authors review the various printing and post-printing processing technologies and discuss how engineering and design are deployed to tailor each type of implant to its patient-specific clinical application, highlighting how anatomical and biomechanical considerations impact their development and manufacturing processes to achieve optimum osteoinductive and osteoconductive properties. The article further examines the benefits of 3D printing, such as customizable geometry and porosity, that enhance osteointegration and mechanical compatibility, offering a leap forward in patient-specific solutions. The comparative analysis provided by the authors underscores the unique challenges and solutions in designing cervical, and lumbar spine implants, including load-bearing requirements and bioactivity with surrounding bony tissue to promote cell attachment. Additionally, the authors discuss the clinical outcomes associated with these implants, including the implications of improvements in surgical precision on patient outcomes. Lastly, they address strategies to overcome implementation challenges in healthcare facilities, which often resist new technology acquisitions due to perceived cost overruns and preconceived notions that hinder potential savings by providing customized surgical implants with the potential for lower complication and revision rates. This comprehensive review aims to provide insights into how modern 3D-printed titanium interbody fusion cages are made, explain quality standards, and how they may impact personalized surgical spine care.

## 1. Introduction

There has been stagnation in the material science of interbody spinal fusion implants for many years [1]. In the last decade, new implant designs were offered to better address technical problems in treating spinal instability and deformity surgically [2,3,4,5,6,7,8]. With a shift from image-based medical necessity criteria for spine surgery towards more targeted procedures employing personalized surgical pain management protocols [9,10] not only started the design aspect of interbody fusion implants to change from rigid to geometrically expandable cages but also the material science aspect of it. The manufacturing techniques underwent significant transformation [11,12].

Traditionally, poly-ether-ether-ketone (PEEK) has been used for many decades as the source material for machined cages for interbody spinal fusion because of its high degree of biocompatibility and a modulus of elasticity close to that of corticocancellous bone found abundantly in the vertebral bodies and its anatomical confines—the endplates [12,13]. PEEK’s primary disadvantage lies in its biological inertness, which is responsible for poor osteointegration. The latter can be problematic in the context of failed interbody fusion and poor clinical outcomes. To resolve some of these problems, surface coating with more osteoinductive biomaterials such as calcium phosphates [14] and osteoconductive materials such as titanium coatings [15] have been proposed and clinically tried [16]. While these added bioactive coatings benefited patients in most cases, delamination [17,18] of surface coatings from the PEEK resulted in substantial titanium coating loss in others after impaction created particle debris; this was thought to delay fusion [19]. Composite cages made from an inner PEEK core and outer titanium components were also tried but soon lost traction in the marketplace with the advent of geometrically expandable cages [1]. Various designs expansion mechanisms allowed the introduction of the cage through a minimally invasive approach into the intervertebral disc space employing the posterior- or transforaminal lumbar interbody fusion technique—PLIF [6,20,21,22,23,24] and TLIF [25,26,27], respectively. However, high-stress concentration produced subsidence-related issues which in some patients may cause pain due to non-union or loss of indirect neural element decompression, or loss of deformity or instability correction [7,26,28,29,30,31,32].

The unique material properties of medical grade Ti6Al4V titanium [33], combined with the capabilities of the 3D printing technology [34,35,36], offer unprecedented opportunities to improve patient outcomes in spine surgery. This innovation allows for the creation of mechanically robust interbody fusion devices that can be precisely tailored to each patient’s unique anatomical dimensions and optimized for bioactive properties. This cutting-edge approach has not only elevated the design and manufacturing process of orthopedic and spinal implants but also significantly enhanced their functionality [37,38], customization [39], and overall effectiveness in surgical applications [40] where devices are needed that can reliably maintain the vertebral spacing while facilitating the fusion process between adjacent vertebrae to reduce pain [41].

This article highlights the cutting-edge advancements in 3D-printed titanium interbody fusion cages, focusing on the differing requirements and designs tailored to spinal implant applications. We examined the specific challenges associated with various spinal regions employing the inclusion and exclusion criteria listed in Table 1 to identify the literature relevant to this article.

**Figure 1:**
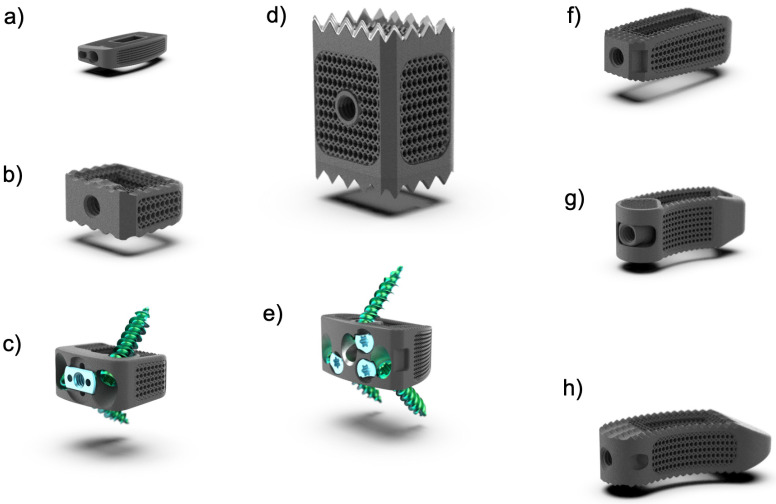
Displayed are eight distinct types of 3D-printed titanium mesh spinal cage implants, each custom-designed for specific spinal surgeries. The implants shown are as follows: (**a**) Lateral Lumbar Interbody Fusion (LLIF) Cage—designed for lateral approach lumbar fusion surgeries, helping to maintain disc height and spinal alignment while encouraging bone growth; (**b**,**c**) Cervical Cages—designed for anterior cervical discectomy and fusion (ACDF) procedures (**a**) with a buttress plate and (**c**) with an integrated buttress mechanism, providing stability and promoting bone growth between cervical vertebrae; (**d**) Corpectomy Cage—applied in corpectomy procedures to replace vertebral bodies removed due to disease or injury, providing structural support and aiding in spinal reconstruction over one or multiple spinal motion segments; (**e**) Anterior Lumbar Interbody Fusion (ALIF) Cage—used for lumbar fusion surgeries, placed through an anterior approach to maintain disc height and alignment while facilitating bone fusion in the anterior spinal column, (**f**) Posterior Lumbar Interbody Fusion (PLIF) Cage—used in posterior lumbar fusion surgeries, facilitating the fusion of the lumbar vertebrae and providing stability; and (**g**,**h**) Transforaminal Lumbar Interbody Fusion (TLIF) Cages—utilized in lumbar fusion surgeries via a transforaminal approach. These 3D-printed titanium mesh cages are engineered for precision and durability, offering enhanced biocompatibility and osteointegration to improve surgical outcomes and patient recovery.

**Figure 2:**
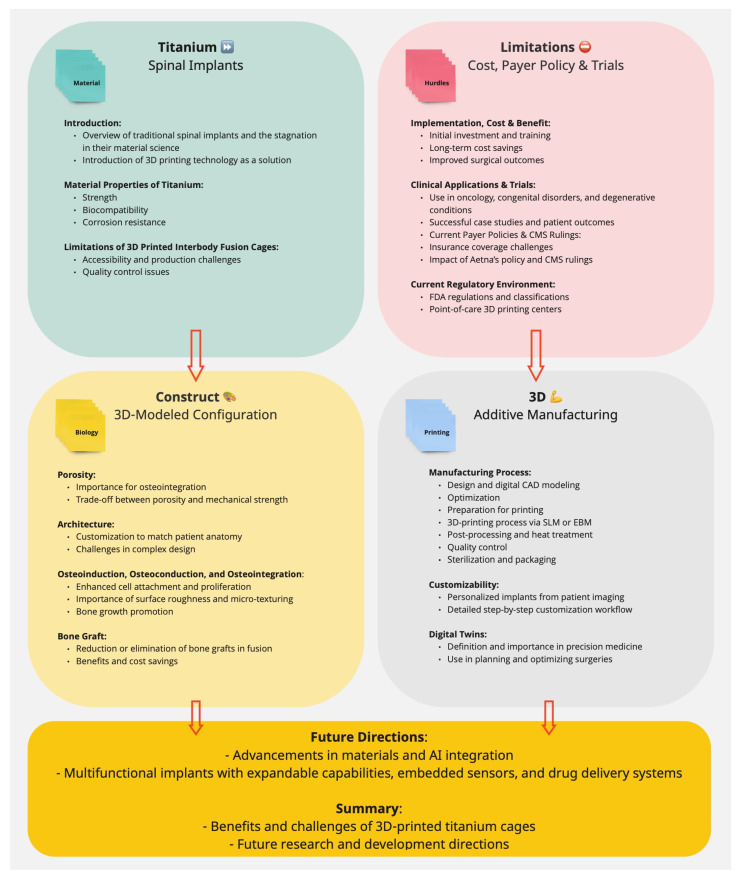
This block diagram outlines our comprehensive approach to incorporating 3D printing technology into spine surgery. It begins with an overview of titanium’s key properties, including strength, biocompatibility, and corrosion resistance, and highlights the role of porosity in osteoinduction, osteoconduction, and osteointegration. The customization process is detailed to match patient-specific anatomy. The step-by-step manufacturing process encompasses design and digital CAD modeling, optimization, preparation, 3D printing via SLM or EBM, post-processing, heat treatment, quality control, and sterilization. These steps aim to enhance surface roughness and micro-texturing for optimal cell attachment and bone growth, thereby eliminating the need for bone grafts. The diagram also highlights clinical trial applications in oncology, congenital disorders, and degenerative conditions, while addressing limitations such as accessibility, production challenges, and quality control issues. It discusses payer policies and CMS rulings, focusing on insurance challenges and the impact of Aetna’s policy and CMS decisions on reimbursement. The regulatory environment is explained, covering FDA regulations and point-of-care 3D printing centers. Future directions include advancements in materials, AI integration, and multifunctional implants with expandable capabilities, sensors, and drug delivery systems. The discussion section summarizes the benefits and challenges of 3D-printed titanium cages and outlines future research directions.

In this article, we emphasize the pivotal role of modern 3D printing technology in the development of patient-specific, anatomically accurate, and biomechanically optimized spinal implants (Figure 1). This technology allows for the creation of implants that precisely match the unique anatomical features of each patient, enhancing surgical precision and outcomes. We also aim to illustrate the transformative potential of 3D-printed titanium cages in significantly advancing spinal surgery, offering detailed insights into current achievements and their implications for the future of personalized spine care. By tailoring implants to individual patient needs, 3D printing technology not only improves the efficacy of surgical interventions but also paves the way for more customized and effective treatments, ultimately benefiting patients with superior, targeted healthcare solutions. Our approach to integrating 3D printing technology in spine surgery is illustrated in Figure 2.

## 2. Material Properties of Titanium

Titanium’s material properties make it an ideal candidate for medical implants [42,43,44,45]. Its strength-to-weight ratio is comparable to that of human bone [46], providing the necessary support without adding excessive weight. Additionally, titanium is highly biocompatible, meaning it does not elicit a significant immune response, and it has excellent corrosion resistance [47], which ensures longevity and durability within the human body. The ability to manipulate titanium using 3D printing technology further enhances its applicability, allowing for the production of complex, customized implants that can meet specific clinical needs. However, the design considerations of porosity, architecture, and strength present advantages and challenges that must be carefully balanced to promote osteointegration and enhanced biomechanical stability [48,49,50].

### 2.1. Porosity

Porosity in titanium implants is a critical factor for promoting osseointegration [51], the process by which bone cells adhere to and grow into the implant surface. This process enhances the stability and longevity of the implant [52]. Porous structures can also reduce the stiffness of the implant, making its mechanical properties more similar to natural bone, which helps distribute the load more evenly and reduces stress shielding effects [53]. While porosity is beneficial for osseointegration, it can compromise the mechanical strength of the implant. Highly porous structures may be more susceptible to fatigue and mechanical failure, especially under the repetitive loading conditions experienced in the spine [54]. Balancing the porosity level to ensure sufficient bone ingrowth while maintaining structural integrity is a critical challenge in designing 3D-printed titanium implants.

### 2.2. Architecture

**Figure 3:**
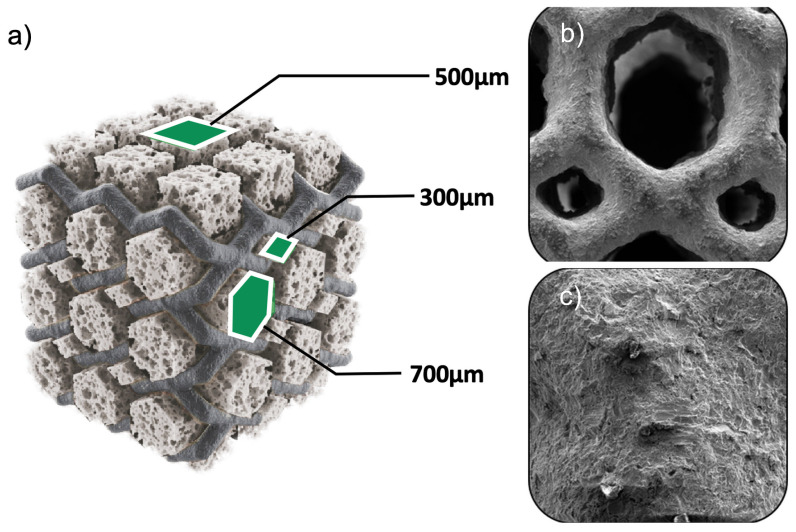
Shown is a 3D-printed titanium mesh model employed in a spinal cage and the result of an SEM analysis. The first image (**a**) shows a Model of a 3D-printed titanium mesh spinal showcasing a proprietary design with a pattern of three distinct pore sizes including hexagonal pores measuring 700 µm in size offering additional stability and support within the mesh, quadratic pores sized at 500 µm, and diamond-shaped pores at 300 µm. These different pores are designed to enhance the mesh’s porosity and osteoconductive properties. The second image (**b**) shows an SEM image at 1000× magnification, which provides a detailed view of the 3D-printed pore pattern at 1000× magnification. This image highlights the precise arrangement and uniformity of the hexagonal and diamond-shaped pores, demonstrating the high-resolution capabilities of 3D printing technology in creating sophisticated mesh structures. The third image (**c**) shows another SEM image at 25,000× magnification revealing the surface morphology of the 3D-printed titanium. This image captures the micro-scale texture and topographical details, showcasing the surface roughness and porosity critical for promoting cell attachment and bone integration. These images collectively illustrate the advanced engineering and precision of fabricating 3D-printed titanium mesh spinal cages, emphasizing their potential to enhance spinal fusion outcomes through optimized pore design and surface characteristics.

The architecture of 3D-printed titanium implants can be precisely controlled to match the complex geometry of the patient’s anatomy [55]. This customization can improve the fit and stability of the implant, leading to better clinical outcomes [56]. Advanced architectural designs, such as interconnected structures, can also enhance the mechanical interlocking with bone tissue and provide vascularization and nutrient flow pathways [57,58], which are crucial for bone health and healing. Designing such complex architectures can be challenging and requires sophisticated modeling and simulation tools (Figure 3) [59]. These more complex designs may also be more difficult to manufacture consistently, potentially leading to variations in implant quality [60]. These need to be monitored with robust quality control measures and could increase production costs. Furthermore, while complex architectures can enhance specific properties, they may also introduce stress concentrators that could lead to mechanical failure under high loads.

### 2.3. Strength

The mechanical strength of titanium, combined with the precision of 3D printing, allows for creation of implants that can withstand significant physiological loads [61]. This strength is crucial for maintaining the spine’s structural integrity and other load-bearing applications. 3D printing enables the fabrication of implants with optimized internal structures that can provide high strength while minimizing weight [62]. Achieving the right balance between strength and porosity can be challenging. Increasing porosity to enhance osseointegration often reduces the overall strength of the implant. Additionally, the anisotropic nature of 3D-printed materials, where properties differ based on the direction of printing, can lead to variability in strength and performance [63]. Ensuring consistent mechanical properties across different implants and manufacturing batches requires rigorous quality control and testing protocols [64,65].

## 3. Manufacturing Process

3D printing, also known as additive manufacturing [66], is transforming the production of spinal interbody fusion cages. The process of 3D printing titanium spinal interbody fusion cages involves several steps, from computer-aided design (CAD) to the final manufacturing and post-processing stages. It involves the following steps:

Design and Digital CAD Modeling: The process begins with creating a digital 3D model of a spinal cage using SOLIDWORKS 2024 SP3.1 CAD software [67]. This model is tailored to the patient’s anatomy, ensuring a precise fit. Engineering strategies are centered around the design of a cage with specific dimensions, pore structures, and surface textures to optimize its performance for the intended application.

1. Optimization: The digital model is optimized for structural integrity, porosity, and mechanical properties [68]. Simulation software can predict how the cage will perform under physiological loads, allowing adjustments to be made before manufacturing.
2. Preparation for Printing: The optimized CAD model is converted into a stereolithography (STL) file, a format widely used in 3D printing [69]. This file contains detailed instructions for the printer, including layer-by-layer construction data. The STL file is then processed by slicing software, which divides the model into thin horizontal layers. Each layer represents a cross-section of the final object and provides the 3D printer with a roadmap for constructing the cage.
3. 3D-Printing Process via Selective Laser Melting (SLM) or Electron Beam Melting (EBM): These are the two primary methods used for 3D printing titanium implants. In SLM, a high-powered laser selectively fuses titanium powder layer by layer according to the digital design [70]. The powder is spread evenly on a build platform, and the laser melts the powder in precise locations to form each layer of the cage. The type of laser typically used is a fiber laser or a Ytterbium (Yb) fiber laser [71]. These lasers are commonly employed in SLM or Direct Metal Laser Sintering (DMLS) processes. Fiber lasers used for metal 3D printing generally operate at a wavelength of around 1.06 μm (1060 nm), which is effective for melting titanium powder [72]. The power output of these lasers can range from 200 watts to several kilowatts, depending on the specific requirements of the printing process and the thickness of the material being processed [73]. Fiber lasers provide a high-quality, focused beam that allows for precise melting and solidification of the titanium powder, which is crucial for creating detailed and accurate components. Similar to SLM, EBM uses an electron beam instead of a laser to melt and fuse titanium powder. This method is performed in a vacuum environment, which can be beneficial for certain material properties. The typical voltage range for the electron beam in EBM machines is between 60 kV and 150 kV [74]. This high voltage accelerates electrons to high speeds, creating the kinetic energy necessary to melt the metal powder. Electromagnetic lenses focus the electron beam to a fine point, allowing precise control of the beam diameter and spot size [75]. The process differences for SLM and ELM are summarized in Table 2.
4. Layer-by-Layer Construction through Layer Deposition: The printer lays down a thin layer of titanium powder on the build platform [76]. The laser or electron beam then melts the powder according to the sliced model’s specifications. This process is repeated for each layer until the entire cage is constructed. The printer follows the design’s intricate details, including any internal lattice structures or porosity features, to ensure optimal performance.
5. Post-Processing and Heat Treatment: After printing, excess titanium powder is removed. This powder can be recycled for future prints, minimizing waste. The printed cages undergo heat treatment [77] to relieve any residual stresses from the printing process and enhance their mechanical properties [78]. Additional surface treatments, such as sandblasting or polishing [79], may be applied to achieve the desired surface texture and improve osseointegration.
6. Quality Control: The finished cages undergo rigorous inspection and testing to ensure they meet all necessary standards and specifications. This includes dimensional checks, mechanical testing, and biocompatibility assessments.
7. Sterilization and Packaging: The cages are sterilized to ensure they are safe for surgical implantation. Standard sterilization methods include gamma irradiation, ethylene oxide gas, or autoclaving [80]. The sterilized cages are carefully packaged to maintain their sterile condition until they are ready for use in surgery.

This manufacturing process facilitates customization to tailor each implant to a patient’s specific anatomy and enhances the fit and performance of the cage while combining a high strength-to-weight ratio and corrosion resistance with optimized complex pore structures that promote bone ingrowth and osseointegration. Additive manufacturing minimizes material waste compared to traditional subtractive manufacturing methods where implants are CNC (Computer Numerical Control) machined from titanium stock [81]. At the same time, there may be higher initial setup and production costs of 3D printing as with any new technology. However, these may be offset by material savings and reduced downstream costs on the clinical side due to higher customization and reduced surgical revisions [82].

## 4. Osteoinduction, Osteoconduction, and Osteointegration

3D-printed spinal interbody fusion cages with a porous architecture are designed to enhance osteointegration, the process by which bone tissue forms and integrates with an implant. These cages often consist of intricate pore structures between 300 to 700 µm and may have diagonal-shaped [83,84], square-shaped or hexagonal-shaped pores. These varying pore shapes and sizes are strategically designed to support both osteoinduction and osteoconduction, which are critical for successful spinal fusion.

**Figure 4:**
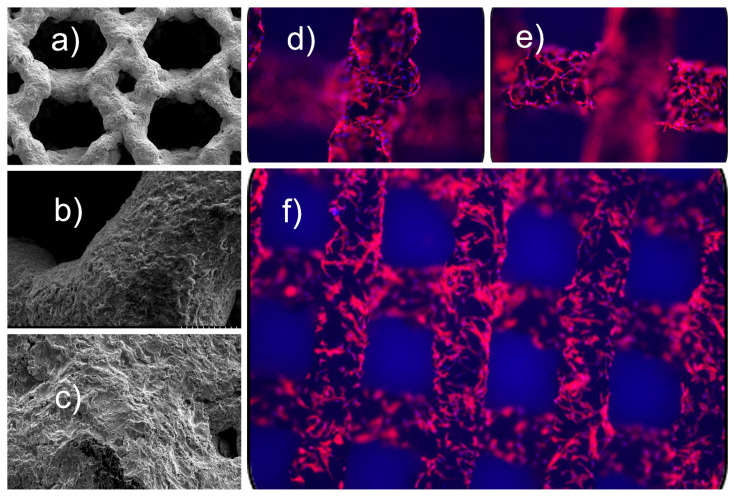
Shown are SEM images of (**a**) hexagonal pores measuring 700 µm in size with interlaced diamond-shaped pores at 150× magnification, (**b**) 300× magnification of the same hexagonal pore and (**c**) a 2000× magnification of the lattice beams whose surface roughness, micro-texturing, and micro-pores typically in the range of a few micrometers play crucial roles in promoting the attachment, proliferation, and differentiation of bone-forming cells. These micropores further increase the surface area and provide additional sites for cellular attachment and ingrowth. The surface roughness appears irregular and textured, with a mix of peaks and valleys, ridges, and grooves resulting from the layer-by-layer additive manufacturing process. These micro-textures create a conducive environment for osteoblasts and other bone cells to adhere more firmly. Depending on the printing direction and parameters, directional patterns may be visible, guiding cellular orientation and spreading, which is beneficial for organized tissue growth. At 2000× magnification, nanoscale roughness can be observed, which provides additional anchoring points for cells and proteins. These tiny irregularities help in the initial cell adhesion processes by increasing the contact points between the cell membrane and the implant surface. The presence of an interconnected porous network allows for better nutrient and waste exchange, facilitating cellular metabolism and promoting a healthy environment for cell proliferation. Titanium also has a surface chemistry characterized by an oxide layer. Titanium naturally forms a thin oxide layer on its surface, which is visible under SEM as a uniform coating ©. This oxide layer enhances biocompatibility and supports protein adsorption, which is critical for cell adhesion. The surface energy of the titanium beams is influenced by their micro-texture and chemistry, making them more hydrophilic. This hydrophilicity is beneficial as it improves the wettability of the surface, allowing bodily fluids to spread more easily, enhancing protein adsorption, and facilitating cell attachment. Occasionally, small particles of unmelted or partially melted titanium powder might be adhered to the surface. These particles can provide additional micro- and nano-scale topographical features that aid in cellular attachment. (**d**–**f**) MSC differentiation and morphology are dependent on the surface topography of the device. An in-vitro analysis of cellular differentiation based on surface roughness and coating was performed with fluorescence microscopy images of MSCs cultured on 3D titanium scaffolds for 5 days. Cells were stained with F-actin (red) and nuclei with DAPI (blue) revealing a favorable response of primary mesenchymal stem cells to the 3D architecture of the printed titanium scaffolds eliciting an osteogenic response with different surface treatments.

Cell attachment and proliferation may occur within the 3D, porous lattice structure inherent to many 3D-printed titanium interbody spinal fusion cages. Osteoblasts and other bone-forming cells attach to the surface of the titanium scaffold. The rough surface texture created by the 3D printing process enhances cell attachment. Once attached, these cells proliferate and begin to produce extracellular matrix (ECM), which provides the framework for new bone formation (Figure 4).

Osteoconduction refers to the ability of a scaffold or implant to support the growth of new bone along its surface and within its pores [85]. The 3D-printed porous architecture of the spinal interbody fusion cages plays a crucial role in this process. The combination of 300-micrometer diagonal-shaped pores, 500-micrometer square-shaped pores, and 700-micrometer hexagonal-shaped pores provides an optimal environment for osteoconduction [86,87,88,89]. The interconnected pore structure increases the surface area available for bone growth, facilitating the migration and attachment of osteogenic cells [90].

Osteoinduction refers to the process by which osteoprogenitor cells are stimulated to differentiate into osteoblasts, the bone-forming cells [85]. This process involves several key components. The porous architecture of the implant facilitates the infiltration of MSCs from the surrounding bone and bone marrow. These cells migrate into the pores and, under the influence of BMPs and other growth factors, differentiate into osteoblasts. Bone Morphogenetic Proteins (BMPs) are a group of growth factors and cytokines that play a pivotal role in osteoinduction. BMPs bind to receptors on the surface of mesenchymal stem cells (MSCs) and induce their differentiation into osteoblasts. The surface characteristics of the 3D-printed titanium cages including surface roughness, micro-texturing and pores, hydrophilicity, and many others impact MSCs differentiation into other types of cells (Table 3). Conceivably, this process could be aided by coating with or impregnating the 3D-printed device with BMPs to enhance this process. Other growth factors such as transforming growth factor-beta (TGF-β), insulin-like growth factors (IGFs), and fibroblast growth factors (FGFs) may also be relevant in this process as they contribute to the induction of osteogenesis by promoting the proliferation and differentiation of MSCs.

The combined effects of osteoinduction and osteoconduction lead to osteointegration—the seamless integration of the implant with the surrounding bone tissue [85]. As osteoblasts produce extracellular matrix (ECM) and mineralize, new bone tissue gradually replaces the scaffold. Over time, the implant becomes an integral part of the spinal structure, restoring stability and function.

Specifically, osseointegration begins with the absorption of water molecules, proteins, and lipids onto the implant surface [91,92]. The host response is specifically influenced by the properties of these proteins [92]. Proteins like fibronectin and vitronectin often trigger inflammation by facilitating platelet attachment [92,93,94]. This attachment leads to the formation of a fibrin clot, creating a meshwork architecture that aids cell migration toward the implant surface [95,96]. Neutrophils and macrophages use this mesh to eliminate pathogens and necrotic tissue [97,98].

Mesenchymal stem cells (MSCs) that reach the implant surface encounter inflammatory cytokines, which affect their differentiation into osteoblasts, chondrocytes, and fibroblasts [99,100]. Gittens et al. found that MSCs in this environment tend to form bone and soft tissue, although the implant’s characteristics also play a role in this process [95].

Once the fibrin meshwork is established, bone can develop on the bone surface near the implant (distance osteogenesis) and directly on the implant surface (contact osteogenesis) [101]. Osteoblasts at these sites may proliferate for several generations or lay down proteins to form the lamina limitans, also known as the cement line [101,102,103]. This protein-rich layer encourages further osteoblast migration and maturation.

Subsequently, bony remodeling occurs as osteoclasts resorb newly formed bone to repair microcracks and prepare the surface for new bone formation [104]. This process creates resorption lacunae with a nano-topography that signals osteoblasts that the surface is ready for new bone formation [95]. If the surface properties are inadequate, migrating cells may form fibrous tissue between the implant and bone, leading to bone degradation and implant loosening [105].

## 5. Bone Graft

Traditional fusion procedures often rely on bone grafts placed within a dedicated chamber in the implant to facilitate bone growth and achieve successful fusion. However, the advent of porous 3D-printed titanium cages challenges this necessity, particularly due to their potential to reduce or eliminate the need for bone grafts. These cages, particularly those designed without a bone graft chamber, foster intimate contact between the bone and the implant at the interface [106]. The intricate porous structure of the titanium provides an extensive surface area that promotes bone ingrowth and integration directly with the implant. This direct bone-to-implant contact maximizes the surface contact area between the implant and the vertebral endplates, potentially enhancing the stability and predictability of the fusion process [107].

One theoretical advantage of this approach is the elimination of complications associated with bone grafts, such as resorption and the risk of non-union. Bone grafts sometimes fail to integrate properly, leading to incomplete fusion and additional surgeries [108]. In contrast, porous 3D-printed titanium cages without a bone graft chamber may offer a more reliable and consistent fusion, as the fusion process is not dependent on the performance of the bone graft [106,107,109]. This could result in more predictable and reliable outcomes, reducing non-union incidence and the need for revision surgeries. Eliminating the need for bone grafts also presents the prospect of significant savings [109]. The costs associated with harvesting autografts or purchasing allografts can be substantial, not to mention the additional surgical time and resources required for their procurement or placement. By relying solely on the porous structure of the titanium implant to facilitate fusion, these expenses are effectively reduced, making the procedure more cost-effective [109].

Empirical evidence supports the viability of this approach in clinical practice [107]. Early clinical outcomes without a graft have shown promising results, with successful fusion rates comparable to or even surpassing those of traditional methods involving bone grafts [107,109]. Further clinical studies are needed to verify and validate these observations comprehensively.

## 6. Customizability

3D-printed titanium cages are emerging as strong competitors to expandable lumbar interbody fusion cages by offering a next-level advancement in personalized spinal implants [110]. Unlike expandable cages adjusted intraoperatively to approximate the patient’s anatomy, 3D-printed titanium cages are precisely contoured preoperatively to match the anatomical specifications derived from detailed imaging. This personalized approach ensures a perfect fit, enhancing stability, promoting optimal bone integration, and ultimately improving surgical outcomes.

Customizing 3D-printed spinal interbody fusion cages represents a transformative leap in spinal surgery that could offer a highly personalized approach to treating spinal instability, disc degeneration, and misalignment. This process begins with a thorough preoperative assessment using advanced imaging techniques, including CT, MRI, and plain film X-rays [55,111,112]. Each imaging modality provides critical insights into the patient’s unique spinal anatomy, essential for designing a tailored interbody fusion cage. For example, a CT scan generates detailed cross-sectional images and sagittal and coronal reformats of the spine. These high-resolution images capture the precise dimensions of the vertebrae and intervertebral discs, providing essential data on bone density and the exact measurements of the vertebral bodies. This information is crucial for understanding the bony architecture and topography of the endplates, thus ensuring that the interbody cage will fit perfectly within the intervertebral space. Concurrent MRI scans are often available to visualize the soft tissue structures of the spine, including the intervertebral discs, nerves, and ligaments, to deduce any cage design modifications dictated by the location of the neural elements or other constricting factors related to deformity or instability. Therefore, the MRI data complement the CT images by offering a detailed view of the soft tissue relationships to the bony anatomy. This is particularly important for assessing the condition of the discs and identifying any nerve compressions or soft tissue abnormalities that need to be addressed during surgery [112]. Plain film X-rays may aid in understanding rigid versus dynamic deformity and instability when taken in various positions, such as standing, flexion, extension, or bending. These images further help assess spinal curvature (such as scoliosis or kyphosis). The X-rays provide a broader perspective on the spinal alignment and are integral in planning the corrective measures needed to restore normal anatomy [112].

**Figure 5:**
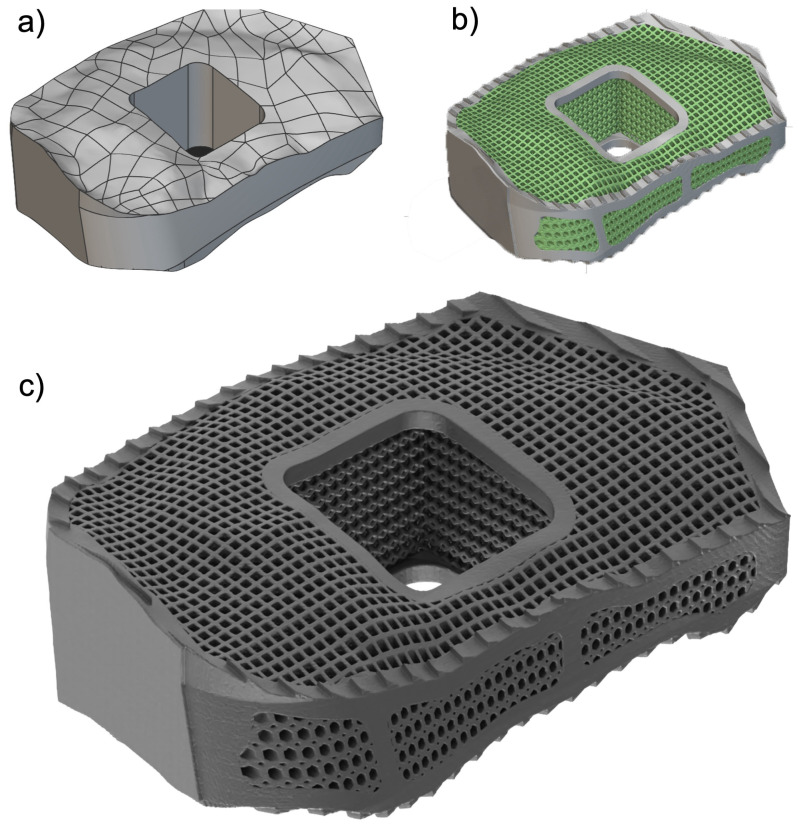
The figure shows the workflow for creating personalized surgical plans and implants. This begins with patient imaging (**a**), such as CT scans and other modalities, which is critical for creating personalized surgical plans and patient-specific implants and instruments. The imaging data is digitally transmitted via a HIPAA-compliant secure portal to the manufacturer. Utilizing this data, along with surgeon inputs and preferences, the manufacturer segments the imaging data using advanced digital technologies, generating personalized surgical plans and implant designs (**b**). The 3D data, representing the bony anatomy, is used to create 3D geometry, which is then converted into 3D CAD models for implant design (**c**). Upon surgeon approval of the surgical plan and device design, the manufacturer uses proprietary processes and printing technology to produce the 3D-printed implant. The implants are sterile-packaged in-house for a short and easily manageable chain of custody and delivered to the healthcare facility in time for surgery. This streamlined “just-in-time” process reduces the timeline to five days compared to the typical six to eight weeks. Finally, the patient receives their personalized, patient-specific implant(s), facilitating a customized recovery process.

Once all imaging data are acquired, they are integrated into specialized software to create a comprehensive 3D model of the patient’s spine. This model precisely represents the patient’s unique spinal anatomy, incorporating both bony and soft tissue structures. With this model, engineers can begin the meticulous process of designing a custom interbody fusion cage. The customization process involves tailoring the cage to fit the exact topography of the intervertebral space. The cage’s shape is designed to match the specific contours and dimensions of the space that it will fill, ensuring a snug fit with the endplates to provide optimal support (Figure 5). The design aims to restore the normal disc height, critical for relieving nerve compression and maintaining proper spinal biomechanics. Additionally, the cage is crafted to correct misalignment, ensure proper sagittal and coronal balance, and address any instability in the spine. The general step-by-step workflow is as follows:

Step 1—Scan: Patient imaging, such as CT scans and other modalities. This is a critical and essential input for creating personalized surgical plans and patient-specific implants and instruments. The imaging data is securely transmitted digitally to the manufacturer via a HIPPA-complaint secure portal.
Step 2—Design: The manufacturer utilizes the patient imaging data, surgeon inputs, and preferences. This segments the imaging data using advanced digital technologies, such as semi-automated or automated imaging segmentation. This process generates personalized surgical plans and implant designs. The 3D data, representing the bony anatomy, is identified and used to create 3D geometry, which is then converted into 3D CAD models for implant design.
Step 3—Manufacture and Deliver: Upon surgeon approval of the surgical plan and device design, the vendor manufactures the 3D-printed implant using their proprietary processes and printing technology. Ideally, the implants are sterile-packaged in-house for a short and easily manageable chain of custody and delivered to the healthcare facility in time for surgery. This can result in a timeline of five days compared to the typical six to eight weeks if the entire design, manufacturing, and sterile packaging process can be handled by one vendor in-house.
Step 4—Surgery: The patient receives their personalized, patient-specific implant(s). This facilitates a customized recovery process. Each custom 3D-printed device should be labeled with a patient identifier, the intended use of the device, the device’s design iteration number, and an expiration date.

## 7. Digital Twins

Customization is further aided by digital twins, which are virtual models mirroring physical objects or systems. They are transforming various fields, including spinal implants. Originating in engineering, they have been applied to complex systems like airplanes and manufacturing. Health digital twins are virtual counterparts of patients, created from genetic information, multimodal patient data, and real-time updates [113]. These models drive precision medicine by providing comprehensive representations of an individual’s health, allowing for simulation and analysis of treatments, disease progression prediction, and tailored interventions.

Digital twin technology leverages Cyber-Physical Systems (CPS) [114] and Closed-Loop Optimization (CLO) [115] to revolutionize healthcare. CPS uses artificial intelligence (AI) and Internet of Things (IoT) technology to synchronize real-time data between physical and digital twins, mimicking human reasoning with big data. CLO enables continuous health monitoring, diagnosis, disease prediction, and treatment optimization for better outcomes.

These virtual models simulate disease progression using patient-specific data, allowing for accurate predictions and tailored treatment responses. Additionally, digital twins employ strategies such as genomic analysis, clinical trial simulation, and continuous health monitoring to support proactive healthcare interventions and preventive measures [116], which are all crucial for chronic disease management. They also provide virtual health coaching, offering personalized lifestyle guidance based on individual health data and goals, and facilitate risk assessment to identify potential health issues early, enabling timely intervention [117].

Furthermore, digital twins aid in surgical planning by providing 3D representations of anatomical structures, allowing for precise planning and simulation of surgeries, reducing risks, and improving accuracy [118]. Specifically, for spinal implants, digital twins can predict the biomechanical properties of the lumbar spine under various postures, enhancing spine treatment planning and surgical optimization.

Digital twins have the potential to significantly impact 3D printing technology in the development and optimization of spinal implants, such as interbody fusion cages by creating highly accurate virtual models of a patient’s spine using data from imaging technologies like MRI and CT scans as discussed above in the customizability section. Digital twins can simulate the cage’s behavior within the patient’s body before printing by performing stress testing, load-bearing analysis, and other biomechanical assessments to ensure the implant will function as intended under various conditions. This predictive capability helps in refining the design and improving the implant’s performance, and durability, identifying the best combinations for strength, flexibility, and biocompatibility. This process can lead to the development of implants that are not only more effective but also more efficient to produce.

Digital twins allow surgeons to plan and rehearse the implantation procedure in a virtual environment. By visualizing the exact placement and alignment of the fusion cage, surgeons can minimize intraoperative uncertainties and enhance the precision of the surgery, potentially leading to better outcomes and faster recovery times for patients. The surgical team may use the 3D model to simulate the operation with hands-on bio-models where the surgeon can assess the fit of the cage using a replica of the patient’s spine [119], planning the optimal placement and fit of the custom cage [120,121]. Mobbs et al. [122] and Ling et al. [123] also reported positive outcomes in lumbar and laminoplasty surgeries, respectively, using 3DP models for preoperative planning. This pre-surgical planning increases the precision with which the procedure can be carried out through the desired anterior, posterior, or lateral approach into the intervertebral space, reducing the risk of complications and improving the likelihood of a successful clinical outcome. The precise fit of the cage provides immediate stability.

Virtual twins may also be useful in postoperative monitoring to make adjustments after the implant is in place with updated real-time data from the patient. Another benefit may be reduced development time and costs by enabling thorough testing and optimization in a virtual environment, digital twins can streamline the development process for 3D-printed spinal implants. This efficiency reduces the time and cost associated with bringing new implant designs to market. They may also facilitate collaboration between multidisciplinary teams, including engineers, surgeons, and researchers, by providing a comprehensive and interactive model to work on.

## 8. Implementation, Cost & Benefit

Implementing 3D-printed titanium cages in spinal surgery, like any other new technology, faces several hurdles when introducing this technology into healthcare facilities [124]. One of the primary challenges is the initial investment in advanced 3D printing technology, including regulatory approval [64], after meeting all safety and efficacy standards on the vendor’s side, which may be passed on to hospitals and ASCs. Further, there are initial training requirements for surgeons and operating room staff to utilize this new approach effectively. The integration of this technology into existing medical practices requires a shift in both infrastructure and mindset, moving from traditional mass-produced implants that require many surgical trays of instruments to customize the spinal construct intraoperatively to highly personalized, patient-specific solutions where intraoperative customization is minimal since it has already played out on the frontend of the operation during the initial planning phase.

Despite these implementation hurdles, a future cost-benefit analysis may reveal significant advantages of 3D-printed titanium cages over traditional interbody fusion systems. Vendors are likely to pass on the long-term cost savings inherent in the additive manufacturing process characterized by reduced material waste, driven by their vested interest in ensuring the successful implementation of this technology. The upfront costs—if higher—may be offset by several indirect cost savings. One notable benefit is the reduced complexity and quantity of instrumentation required during surgery. Traditional systems often involve multiple trays of instruments that must be sterilized, prepared, and managed throughout the procedure, thus increasing care costs due to higher staffing requirements and higher in-house resource utilization. In contrast, the personalized fit of 3D-printed cages reduces the need for extensive intraoperative customization and delivery to the healthcare facility using “just-in-time” production, streamlining the surgical process and reducing the number of instruments needed. In the future, 3D-printed cages can be paired with disposable instruments, further lowering costs per case. This combination reduces the financial burden of sterilization and instrument maintenance and enhances operational efficiency. Disposable instruments are particularly advantageous in ambulatory surgery centers (ASCs), where resources and infrastructure may be more limited than in large hospitals. By minimizing the need for complex setups and specialized equipment, surgeries can be performed more efficiently and safely in ASCs, offering more efficient high-quality care in a cost-effective setting which has been shown to translate into better patient outcomes in other areas of spinal and orthopedic surgery [124,125]. The improved fit and integration of the 3D-printed titanium implant into existing protocols can lead to improved workflow in the operating room, faster recovery times, reduced complication rates, and fewer revision surgeries. These factors benefit patients by improving their quality of life and alleviating the financial strain on healthcare systems by reducing the long-term costs associated with postoperative care and complications.

## 9. Clinical Applications & Trials

The application of 3D-printed implants in spinal surgery demonstrates significant promise across various conditions (Table 4). The literature on 3D-printed implants primarily addresses their application in oncological spine pathology. Xiao et al. [126] highlighted the use of 3D models in preoperative planning for “en bloc” resection of cervical spine tumors. Ahmed et al. [127] similarly illustrated the utility of 3D models for intraoperative reference, achieving negative margins. The technology has also been applied in reconstructing deformities post-tumor excision. For instance, Wei et al. [128] used a 3D-printed sacral replacement prosthesis following sacral chordoma resection, observing asymptomatic instrumental failure and bone-prosthesis ingrowth after eight months. Xu et al. [129] utilized a 3D-printed implant for axial vertebral body replacement after C2 Ewing sarcoma surgery, reporting no complications and satisfactory imaging at one year. Customized implants like these achieve superior fit and retain motion. Li et al. [130] created a self-stabilizing 3D implant for multilevel vertebral body reconstruction post-thyroid carcinoma resection, with no subsidence at one year. Kim et al. [131] and Choy et al. [132] also reported successful outcomes with 3D-printed implants for sacral osteosarcoma and hemangioendothelioma, demonstrating the potential for no complications and improved patient symptoms using 3D-printed impacts.

In congenital disorders, 3D-printed implants have shown significant promise as well. Mobbs et al. [124] used them for congenital defects, achieving positive outcomes. Yang et al. [135] conducted a study on adolescent idiopathic scoliosis patients, finding that 3D-printed bio-models reduced operative time, blood loss, and screw misplacement, especially in severe cases. Tu et al. [136] reported high accuracy in screw placement and no neurovascular complications in ankylosing spondylitis patients using 3D-printed osteotomy guides.

Degenerative disorders of the spine, highly prevalent and often resulting in chronic pain, also benefit from 3D-printed solutions. Siu et al. [139] reported successful use of 3D-printed titanium cages in an elderly patient with osteoporotic fractures, leading to rapid ambulation and symptom resolution. Rosenzweig et al. [137] demonstrated the potential of 3D-printed scaffolds for promoting osseointegration through in vitro experiments, suggesting broad applicability in degenerative conditions.

**Figure 6:**
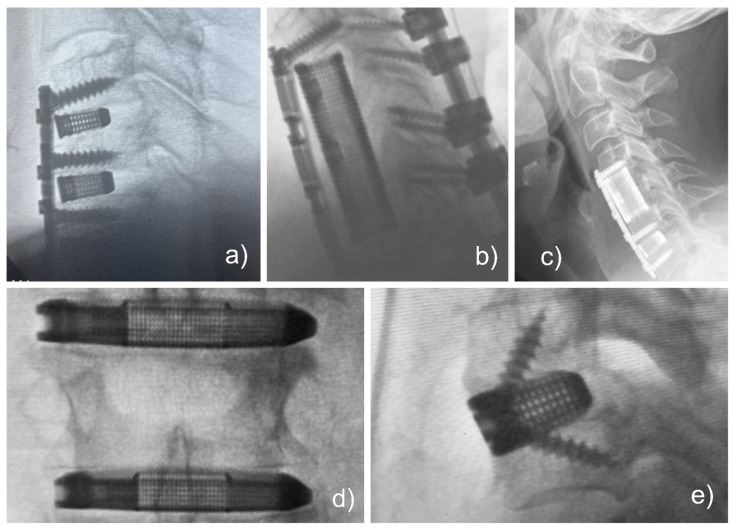
Various clinical applications of 3D-printed custom titanium cages are shown, supported by intraoperative fluoroscopy images. The examples showcase the versatility and effectiveness of these implants in different spinal surgeries: (**a**) three-level anterior cervical discectomy and fusion (ACDF), (**b**) multilevel cervical corpectomy stabilized with a buttress plate and posterior supplemental fixation, (**c**) hybrid cervical corpectomy with ACDF stabilized with a buttress plate, (**d**) lateral lumbar cages placed through a retroperitoneal approach, and (**e**) a single-level ACDF. These intraoperative fluoroscopy images highlight the diverse clinical applications of 3D-printed custom titanium cages, showcasing their adaptability and effectiveness in various spinal surgical scenarios.

Spinal fusion surgeries, essential for correcting spondylosis and deformities, also see significant improvements with 3D-printed technology (Figure 6). Mokawem et al. [142] reported excellent fusion success and improved patient outcomes using 3D-printed titanium cages in lumbar fusion surgeries. Lu et al. [138] demonstrated similar success in cervical corpectomy patients. Thayaparan et al. [140] highlighted the efficacy of 3D-printed patient-specific screws in atlantoaxial fixation, showing no complications or implant failures. Choy et al. [141] reviewed successful cases of anterior cervical decompression using patient-specific 3D-printed implants.

3D-printed cages, particularly those with body lattice structures and microporous endplates, offer significant advantages in reducing subsidence in non-segmental and segmental instrumented transforaminal, [143] lateral [136] and posterior lumbar interbody fusions, as well as in stand-alone fusions [144]. These design features effectively reduce the cage’s stiffness, thereby mitigating the risk of stress shielding and promoting early fusion [83] The porous titanium architecture of these cages not only supports bone ingrowth and segmental mechanical stability but also increases the available space for bone bridging, which is crucial for successful spinal fusion. Clinical studies have shown that titanium cages exhibit a significantly lower overall subsidence rate compared to polyetheretherketone (PEEK) cages [145], with a notably lower incidence of high-grade subsidence. This reduction in subsidence is attributed to the titanium cages’ ability to mimic the modulus of elasticity of native vertebrae, reducing stress at the bone-hardware interface [146]. These findings suggest that 3D-printed porous titanium cages present an effective solution to enhance spinal fusion outcomes while minimizing the risks associated with increased subsidence. While cage fractures are very uncommon, they have been reported in one FDA trial, notably involving the Class 2 device recall of the Stryker Tritanium Posterior Lumbar cage used in TLIF and PLIF procedures. The instances of cage breakage may be partly attributed to the increased porosity of the 3DP cages compared with traditional ones [147].

There is a need for more information on recent experimental and numerical studies in this emerging field of 3D-printing since the current clinical evidence regarding the efficacy and safety of both patient-specific (PS) and off-the-shelf (OTS) 3D-printed spinal implants remains limited. A recent systematic review, adhering to PRISMA guidelines, evaluated peer-reviewed articles and identified twenty-two eligible studies and one book chapter [122,129,130,131,132,133,138,139,140,142,148,149,150,151,152,153,154,155,156,157,158,159,160,161,162]. These studies primarily consisted of case reports and series, focusing on PS designs made from titanium alloys for surgical reconstruction in cases of neoplasia, infection, trauma, or degenerative spinal conditions that present significant anatomical challenges. While the reviewed literature demonstrates the considerable utility of 3DP spinal implants in managing complex spine pathologies and reports positive outcomes from both surgeons and patients, the conclusions drawn are still tentative. This is due to relatively short follow-up periods and a lack of high-powered studies. To advance the clinical use of 3DP spinal implants, there is a critical need for more experimental and numerical studies. These studies should aim to provide standardized reporting of clinical, radiographic, and biomechanical outcomes, thereby enhancing the robustness of the evidence supporting the use of 3DP technology in spinal surgery.

## 10. Limitations of 3D Printed Interbody Fusion Cages

Although 3D-printed spinal implants are becoming more prevalent, they are not yet universally accessible. Only a few vendors possess the necessary printers, skilled labor, and expertise to produce these implants. Mastery of the technical skills and consistent quality is currently limited to a few industry leaders, highlighting issues with printing speed, resolution, and reproducibility [163]. The production of 3D-printed interbody fusion cages for spine surgery can take 10–12 h, plus additional time for post-processing procedures such as heat treatment, residue removal, packaging, and sterilization. This results in a total turnaround time ranging from several days to weeks. These logistical challenges need to be resolved to make custom implants more readily available, a task likely manageable only by the leading industry players.

## 11. Current Payer Policies & CMS Rulings

As with any other new technology, authorization requests for coverage of personalized 3D-printed implants may see some pushback. The technology may get caught up in a recent policy decision by Aetna, the third largest insurance carrier in the US with an 11% market share, currently rations the provision of cervical cages [164]. This policy applies downward pressure on current cervical arthrodesis practices. This decision also disregards patient-consumer choice, the diversity of cultural preferences, and surgeon preferences to achieve the best clinical outcomes.

Aetna’s policy distinguishes between synthetic cages and structural allografts. Under Aetna’s coverage policy, CPT code 20931, used for reporting the placement of structural allografts during cervical arthrodesis, is limited to a single use, regardless of the number of allografts required in the same surgical session. In contrast, CPT code 22853, designated exclusively for cage use, offers higher reimbursement and may be reported for each interspace where a cage is employed. This discrepancy creates a financial disincentive for surgeons to choose synthetic cages, as some payers, including Aetna, consider “spine cages NOT medically necessary for cervical fusion”.

This policy incentivizes the use of allografts, which may only sometimes be the most appropriate choice, and puts customization aspects straight back into the hands of the surgeon in the operating room. This issue extends to using 3D-printed interbody fusion cages, and this policy compromises the doctor–patient relationship and the overall quality of care patients receive. Under Aetna’s current policy, the financial and administrative barriers to using synthetic cages, including 3D-printed implants, are substantial, further limiting the potential for incorporating cutting-edge technology into patient care, thus, effectively stifling innovation. Under these restrictive policies, patients will not receive a 3D-printed cervical cage or a simple synthetic monolithic cage.

Since 2001, the Centers for Medicare & Medicaid Services (CMS) have permitted manufacturers of innovative technologies to apply for a New Technology Add-on Payment (NTAP) designation for their drugs, devices, or diagnostics. This designation provides hospitals with additional payments for using the product during its first 2–3 years on the market. These add-on payments are often critical for hospitals when adopting new technologies. Regardless of the outcome of reorganizing the spine fusion MS-DRGs, CMS should address the resource utilization disparity associated with custom-made anatomically designed spine fusion cages, such as the Aprevo device by Carlsmed^®^ [165,166]. This FDA-designated Breakthrough Technology, with published clinical outcomes, uses personalized planning to create anatomically matched implants for spine fusion patients. The NTAP for procedures using these custom-made cages will end on 30 September 2024. If these procedures are not reassigned to an MS-DRG that adequately covers the necessary resources for FY2025, hospitals will face a financial disincentive to use this innovative technology, limiting access for Medicare beneficiaries. The International Society for the Advancement of Spine Surgery (ISASS) has recently urged CMS in a response letter to the ruling on custom cages to address these procedures’ proposed resource utilization disparity and reassign them to MS-DRGs that reflect their higher cost and resource needs.

## 12. Current Regulatory Environment

The FDA designated 3D printing as a breakthrough technology. While the majority of 3D printed devices are regulated through the FDA’s Center for Devices and Radiological Health (CDRH) [167], the FDA’s Center for Biologics Evaluation and Research (CBER) regulates all biological, cellular, or tissue-based applications of additive manufacturing, the FDA’s Center for Drug Evaluation and Research (CDER) regulates all drug applications of additive manufacturing, and the Office of Combination Products (OCP) regulates products that have components that would typically be regulated by different FDA centers [168].

One point of frequent misunderstanding that the FDA has clarified in past workshops and documents [169,170] is that the FDA does not regulate the use of raw 3D printing materials, final printing materials, or specific printing processes for unspecified uses. Rather, it clears specific devices that may or may not be designed through 3D printing (using existing or novel raw materials) for specific clinical indications. However, in Premarket Notification submissions, it may still be useful to include the names of previously cleared devices that share the same additive manufacturing process to help make the case for substantial equivalence [169].

Recent advances in 3D printing technology have enabled the establishment of point-of-care (PoC) 3D printing centers. These centers blur the distinctions between healthcare providers, medical facilities, and device manufacturers, leading to regulatory ambiguity. In this context, PoC 3D-Printing is the on-demand creation of 3D-printed diagnostic anatomic models, surgical instruments, or other medical devices using a patient’s medical imaging data, either at the point of care (such as a hospital) or at a centralized facility owned by the healthcare organization [171]. This definition specifically focuses on patient-matched devices and does not encompass other types of 3D printing of mass-produced implants such as those made at a manufacturer’s facility.

The FDA is in the process of formulating regulations for those clinical applications where the 3D-printed devices are made in the Point-of-Care (PoC) setting. It has defined three categories: (1) diagnostic use of anatomic models [172], (2) patient-specific surgical instruments [169], and (3) patient-specific implants [169,170]. Diagnostic use anatomic models are the most common, typically derived from patients’ CT imaging, allowing visualization of patient-specific anatomy in three dimensions; particularly in the dental, oro-, and craniomaxillofacial field [173,174]. These models if sold by manufacturers to hospitals usually fall under FDA Class II regulations.

In August 2017, the RSNA SIG and the FDA held a joint session at the FDA White Oak Campus to define the regulatory aspects for PoC diagnostic use anatomic models [172]. It was clarified that diagnostic use for a 3D-printed anatomic model falls under medical device regulations. This meeting established that United States companies manufacturing and selling patient-specific 3D printed anatomic models must adhere to the guidelines set by the RSNA and the FDA. Prior to this session, there was some suggestion in the literature that PoC patient-matched anatomic models could be considered medical image hardcopies and not medical devices [169,175]. This interpretation is no longer valid. Under 21 CFR 892.2040, 3D printers for these models are classified as medical image hardcopy devices.

Patient-specific surgical instruments and implants, which are less common in the PoC setting than diagnostic use anatomic models, typically fall under Class II regulations but may require Class III if they pose unique safety or efficacy concerns or lack a substantially equivalent predicate device [169]. These devices also face regulatory uncertainty, which the FDA has addressed in a discussion paper titled “Discussion Paper: 3D Printing Medical Devices at the Point of Care” released in early 2024 [176].

Legally marketed 3D printed devices must meet the same regulatory requirements as non-3D printed devices, including Quality Systems (QS) regulations [170]. These regulations ensure medical devices consistently meet necessary standards. Although PoC centers face regulatory uncertainty, they must prioritize safety in their manufacturing processes (Table 5).

Although the FDA has not yet published specific regulations for Point-of-Care (PoC) 3D printing centers, discussions with stakeholders, including engineers, the medical device industry, ASME, RSNA 3D SIG, PoC centers, physicians, and surgeons, have begun to develop a framework for future regulations. While specific regulations are pending, PoC centers should adhere to best practices to ensure safety and quality (Table 6).

In 2024, the FDA released a discussion paper titled “Discussion Paper: 3D Printing Medical Devices at the Point of Care” [176] intended for custom but not mass-produced 3D-printed implants for the following three scenarios: (1) Healthcare facilities (HCFs) using a medical device production system (MDPS), (2) Traditional manufacturers co-located at or near a HCF site, and (3) HCFs assuming all traditional manufacturer responsibilities. In dentistry, onsite 3D printing at the PoC is commonplace. To our knowledge, there are currently no onsite 3D printing facilities for spinal implants where the manufacturer is either co-located on or near the HCF’s site. In the hypothetical scenario that such sites existed, the FDA currently stipulates that the traditional manufacturer could use the co-located manufacturing site to 3D print specific devices or implants at the HCF requests. The Traditional Manufacturer with the co-located manufacturing site would be responsible for compliance with FDA regulatory requirements. While this scenario entertained in the FDA 2024 discussion paper may well apply to dental offices where tabletop units coupled with CT-Scanners provide a one-stop solution, it seems far from realistic at present with the industrial-sized printers, postprocessing, and sterilization equipment needed for 3D-printed spinal implants including cages. It is also questionable whether these arrangements would hold up to legal scrutiny if ever challenged. Thus, one should expect several reiterations of FDA regulations about custom 3D-printed spinal implants, including cages.

## 13. Future Directions

The future of personalized spine care is likely to be transformed by the ongoing advancements in 3D printing technology and materials, as well as the integration of artificial intelligence (AI) and machine learning. These innovations are expected to significantly enhance patient-specific implants’ design, customization, and functionality. The continuous enhancement of 3D printing technology is leading to the development of new materials and printing techniques that promise to revolutionize the creation of spinal implants. Future improvements may include using biomimetic materials other than titanium that more closely replicate the biological properties of natural bone, thereby improving biocompatibility and promoting better integration with the patient’s anatomy. Additionally, more sophisticated printing methods could enable the production of implants with complex, patient-specific geometries that are currently unattainable through traditional subtractive manufacturing processes. These improvements are anticipated to result in more durable, effective, and personalized implants, ultimately enhancing patient outcomes and reducing recovery times.

Incorporating AI and machine learning into spinal implants’ design and customization process will also likely impact personalized spine care. AI algorithms can analyze extensive patient data to generate optimal implant designs and surgical plans with unparalleled precision. Machine learning models can continuously improve by learning from prior cases, leading to increasingly personalized and effective treatments. This integration will facilitate a highly tailored approach to spine care, where implants and surgical strategies are meticulously crafted to meet each patient’s unique needs, enhancing the treatment’s overall effectiveness.

A vision for the future of personalized spine care using advanced 3D-printing technologies will likely include the development of multifunctional implants with capabilities that extend far beyond traditional support and stabilization. Key areas of innovation may include:

- Expandable Capabilities: Future implants may be designed with the ability to adapt and expand post-surgery, ensuring a better fit with the patient’s evolving anatomical structures and improving long-term outcomes.
- Embedded Sensors: Advanced implants could be equipped with embedded sensors that provide real-time data on the healing process, allowing clinicians to monitor progress and adjust the treatment plan as needed.
- Drug Delivery Systems: Integrating drug and stimulatory factor delivery mechanisms within the implants could enable the direct administration of medication to the surgical site, promoting faster healing and reducing the risk of infection.

These advancements would represent a convergence of cutting-edge technology and individualized treatment to offer patients superior surgical outcomes and an enhanced recovery experience. The future of 3D printing in personalized spine care is expected to bring technological innovations and AI-driven customization for more effective, patient-centered treatments.

## 14. Discussion

The 3D printing of titanium spinal interbody fusion cages represents a transformative advancement in medical implant technology. By leveraging the precision and versatility of additive manufacturing, these implants can be customized to enhance patient outcomes [182], promote bone growth, and provide durable support for spinal fusion procedures. As this technology continues to evolve, its integration into medical applications will likely expand, offering innovative solutions for complex surgical challenges.

One of the primary advantages of 3D printing technology in the fabrication of titanium implants is its ability to create structures that are structurally sound and tailored to the specific anatomical and mechanical requirements of different spinal regions [183]. The material properties of titanium offer numerous benefits, including excellent biocompatibility, strength, and corrosion resistance [79]—however, the design considerations of porosity, architecture, and strength require attention to detail for the specific spinal application. By carefully balancing these factors, it is possible to create implants that mimic the mechanical properties of human bone [184], promoting better clinical outcomes through enhanced osseointegration and customization. Continued research and development in this field will further refine these technologies and perhaps eliminate the use of adjunctive bone grafts, which brings a whole host of benefits, ranging from reduced direct cost to eliminating any concerns regarding allograft use or donor site pain. Varied pore sizes and shapes [48] are instrumental in promoting osteoinduction and osteoconduction by facilitating the migration, attachment, and differentiation of bone-forming cells and supporting vascularization and nutrient diffusion, creating an optimal environment for new bone growth leading to successful osseointegration crucial for the long-term success of spinal fusion surgeries. As research and technology advance, optimizing these implants will continue to improve patient outcomes and revolutionize spinal surgery. Ultimately, the 3D-printing technology of spinal implants from titanium may lead to more effective and reliable medical implants.

## 15. Conclusions

The 3D printing of titanium spinal interbody fusion cages represents a significant advancement in medical implant technology, leveraging the precision and versatility of additive manufacturing to enhance patient outcomes. These custom implants promote bone growth and provide durable support for spinal fusion procedures by being tailored to individual patients’ specific anatomical and mechanical requirements. The integration of this technology addresses complex surgical challenges and optimizes clinical outcomes through enhanced osseointegration and customization. Varied pore sizes and shapes facilitate osteoinduction and osteoconduction, creating an optimal environment for bone growth. These processes lead to successful osteointegration, which is crucial for the long-term success of spinal fusion surgeries. Despite initial implementation challenges, the benefits—including simplified instrumentation, cost savings from disposable instruments, and improved surgical outcomes—make these implants a compelling advancement in spinal surgery.

## Figures and Tables

**Table 1:** Inclusion and Exclusion Criteria employed in the literature search.

Inclusion Criteria	Exclusion Criteria
3D-printed titanium interbody spinal fusion cages Personalized spine care 3D-printing technology Titanium implants Intervertebral stability Bone growth promotion Spinal alignment restoration Patient-specific clinical application Osteoinductive and osteoconductive properties Customizable geometry Porosity enhancement Osteointegration Mechanical compatibility Cervical and lumbar spine implants Load-bearing requirements Biomechanical properties Cell attachment Surgical precision and outcomes	Non-3D-printed implants Non-titanium materials Non-customized spinal fusion cages Generalized or non-personalized treatments Non-specific spinal surgeries Non-advanced manufacturing techniques Traditional manufacturing processes Non-osteoconductive or non-osteoinductive materials Clinical outcomes without reference to 3D-printed titanium Non-spinal applications of 3D printing

**Table 2:** Key Steps and Characteristics of 3D-Printing Processes via Selective Laser Melting (SLM) and Electron Beam Melting (EBM).

Aspect	Selective Laser Melting (SLM)	Electron Beam Melting (EBM)
Energy Source	High-power laser (usually fiber laser)	High-energy electron beam
Operating Environment	Inert gas atmosphere (usually argon or nitrogen)	High vacuum
Powder Material	Metal powder (e.g., titanium, aluminum, steel)	Metal powder (e.g., titanium, cobalt-chrome, nickel alloys)
Typical Voltage	Not applicable	60 kV to 150 kV
Layer Construction	Layer-by-layer melting and solidification with laser	Layer-by-layer melting and solidification with electron beam
Beam Focus and Control	Optical lenses for focusing and galvanometers for scanning	Electromagnetic lenses for focusing and scanning
Scanning Speed	High, due to the rapid movement of the laser beam	Generally lower than SLM, dependent on electron beam control
Resolution	High, with fine feature capability	High, but slightly less than SLM due to beam spread
Surface Finish	Generally smoother, may still require post-processing	Rougher, often requires post-processing
Build Rate	Moderate to high, depending on laser power and scan strategy	Generally high, due to the high energy density of the electron beam
Material Properties	Excellent mechanical properties; can vary with process parameters	Excellent mechanical properties; very consistent
Typical Applications	Aerospace, medical implants, automotive	Aerospace, medical implants, industrial components
Complexity of Machine	High, requires precise calibration and control	High, requires a vacuum environment and electron beam control
Cost	High, both in terms of equipment and operating costs	High, both in terms of equipment and operating costs
Post-Processing Requirements	May include heat treatment, surface finishing, and removal of support structures	May include heat treatment, surface finishing, and removal of support structures
Powder Recycling	Possible, with careful handling to avoid contamination	Possible, often easier due to the vacuum environment
Build Volume	Limited by machine size, but generally available in various sizes	Limited by machine size, but available in various sizes

**Table 3:** Key factors affecting cell attachment, proliferation, and differentiation of MSCs on titanium surfaces with various micro- and nano-scale features.

Factors	Description
Surface Roughness	Irregular and textured surface with a mix of peaks and valleys, ridges, and grooves from the layer-by-layer additive manufacturing process.
Micro-Texturing	Crucial for promoting attachment, proliferation, and differentiation of bone-forming cells. Creates a conducive environment for osteoblasts and other bone cells to adhere more firmly.
Micro-Pores	Typically in the range of a few micrometers. Increase surface area and provide additional sites for cellular attachment and ingrowth.
Printing Direction and Parameters	Directional patterns may be visible, guiding cellular orientation and spreading, which is beneficial for organized tissue growth.
Nanoscale Roughness (2000× magnification)	Observed nanoscale roughness provides additional anchoring points for cells and proteins, enhancing initial cell adhesion by increasing contact points between the cell membrane and implant surface.
Interconnected Porous Network	Allows for better nutrient and waste exchange, facilitating cellular metabolism, and promoting a healthy environment for cell proliferation.
Titanium Surface Chemistry	Characterized by a naturally forming thin oxide layer, visible under SEM as a uniform coating. Enhances biocompatibility and supports protein adsorption, critical for cell adhesion.
Surface Energy and Hydrophilicity	Influenced by micro-texture and chemistry, making titanium is more hydrophilic. Improves wettability, allowing bodily fluids to spread more easily, enhancing protein adsorption, and facilitating cell attachment.
Small Particles of Titanium Powder	Occasionally present, providing additional micro- and nano-scale topographical features that aid in cellular attachment.
MSC Differentiation and Morphology	Dependent on the surface topography of the device. In-vitro analysis at 5 days shows smooth titanium directs MSC differentiation into fibroblast-like cells, whereas rough surfaces promote osteocyte-like differentiation.

**Table 4:** 3D-Printing applications in spine surgery.

Area	Study	Summary
Oncology	Xiao et al. [126] (2016)	Valuable for pre-operative planning; helps visualize tumor burden and surrounding anatomy
	Xu et al. [129] (2016)	Performed vertebral body replacement after C2 Ewing sarcoma surgery; no complications.
	Kim et al. [131] (2017)	Created a hemi-sacrum post-sacral osteosarcoma resection; no complications; improved symptoms.
	Wei et al. [128] (2017)	Used a sacral replacement prosthesis after sacral chordoma resection; observed asymptomatic instrumental failure and bone-prosthesis ingrowth.
	Choy et al. [132] (2017)	Employed an axial vertebral body device with fixation holes and angled endplates for a T9 primary bone tumor; restored sagittal balance; improved symptoms.
	Li et al. [130] (2017)	Reconstructed multilevel C2–C4 vertebral body after metastatic papillary thyroid carcinoma resection with a self-stabilizing implant; no subsidence at 1 year.
	Mobbs et al. [133] (2017)	Addressed C1/2 chordoma and congenital L5 hemivertebra with occipito-cervical fixation and hemivertebra prosthesis; no imaging abnormalities at 9 and 12 months, despite prolonged operation time.
	Chin et al. [134] (2019)	Achieved posterior instrumentation fixation and osseointegration after en bloc spondylectomy for L1–L3 recurrent giant cell tumor using 3D reconstruction.
	Ahmed et al. [127] (2019)	Referenced 3D models intraoperatively for better tumor visualization and lesion irregularities, minimizing morbidity and achieving negative margins.
Congenital	Yang et al. [135] (2015)	3D technology reduced operative time, blood loss, and transfusion volume without affecting LOS, complication rate, screw misalignment, or radiographic outcome; significantly reduced screw misplacement in patients with Cobb angle > 50 degrees (*p* = 0.02).
	Tu et al. [136] (2019)	Software-aided correction achieved 94% accuracy in screw placement without neurovascular complications.
Degenerative/Decompression/Fusion	Rosenzweig et al. [137] (2015)	In vitro experiments showed successful culturing of primary articular chondrocytes and nucleus pulposus cells on 3D-printed ABS and PLA scaffolds for osseointegration.
	Lu et al. [138] (2017)	3D-printed titanium fusion cage used for anterior cervical corpectomy and fusion in 15 patients with cervical spondylotic myelopathy and OPLL; all patients experienced solid interbody fusion and symptom relief.
	Siu et al. [139] (2018)	Customized cages for lumbar radiculopathy and osteoporosis led to symptom resolution at 6 months and CT-confirmed fusion.
	Thayaparan et al. [140] (2018)	Patient-specific titanium atlantoaxial screws demonstrated successful placement and fixation with no neurological issues or implant failures at a 12-month follow-up.
	Ling et al. [123] (2018)	3D-printed models aided pre-operative planning of “V”-shaped decompressive laminoplasty for multilevel ossification of the ligamentum flavum; successful decompression.
	Choy et al. [141] (2018)	Successful anterior cervical decompression and multi-level fusion with patient-specific 3D-printed titanium implants in complex deformities.
	Mokawem et al. [142] (2019)	Transforaminal or lateral lumbar interbody fusion with silicate-substituted calcium phosphate-packed 3D-printed lamellar titanium cages showed 98.9% fusion success and significantly improved patient outcomes.
	Mobbs et al. [122] (2019)	3D-printed technology in anterior lumbar interbody fusion provided excellent fit, improved pre-operative planning, restored lumbar lordosis, and reduced operative time, leading to clinical improvement.
	Malone et al. [109] (2022)	136 levels in 90 patients. The use of bioactive titanium interbody devices with a large surface footprint results in a high fusion rate despite the use of a small volume of low-cost biological material to lower the economic burden inherent to spinal fusion. 3D-pTi interbody cages without bone grafts outperform PEEK interbody cages with grafts.
	Topps et al. [143] (2023)	90 solid titanium (ST) from 74 patients, and 73 3D-printed (3DPT) interbody levels from 50 patients. both ST and 3DPT cages performed well; however, 3DPT cages were associated with lower rates of subsidence.
	Ham et al. [11] (2023)	31 consecutive patients who underwent single-level posterior lumbar interbody fusion surgery with two 3D-Ti cages with different designs were inserted: a non-window cage on the left side and a window cage on the right side. Using a non-window 3D-Ti cage during lumbar interbody fusion might be acceptable.
	Duan et al. [13] (2024)	Systematic review reporting 9 studies comparing lumbar interbody fusion with 3D-PPT cages versus PEEK cages for lumbar degenerative disease. Compared to the PEEK cage, the 3DPT cage showed a higher fusion and lower subsidence rate. The 3DPT cage may accelerate fusion, and prevent subsidence.

**Table 5:** Quality Systems (QS) regulations for PoC 3D printing centers.

QS-Task	Description
Process Control [170]	Document 3D printer system parameters, such as calibration, maintenance, and environmental conditions. Implement FDA’s cybersecurity guidance if patient data is transferred during the workflow [177]
Imaging Quality [170,178]	Ensure imaging used for modeling devices has sufficient resolution, capturing the smallest anatomy of interest on at least three sequential DICOM images.
Material Documentation [170]	Record chemical names, suppliers, and certificates of analysis for all raw materials, additives, and processing aids. Document the material reuse process to ensure it does not affect device performance.
Support Structures [170]	Detail the use and removal of support structures to ensure they do not negatively impact the final product.
Layering and Meshing [170,178]	Optimize layer thickness for the device’s intended use, ensuring the smallest area of interest is captured on at least three consecutive layers. Document the mesh details of 3D model files to maintain accuracy.
Build Paths	Document the build path and its potential consequences, including fill density and the status of internal voids.
Post-Processing [170,178]	Ensure post-processing steps enhance utility without affecting the device’s intended use or accuracy. Document all post-processing steps and their impact on material properties.
Sterilization [179]	Adhere to ISO standards for sterility and biocompatibility. Document sterility processes and validate that they are compatible with the materials used.
Biocompatibility [179]	Test for biocompatibility of raw materials and the final sterilized product, especially if toxic chemical additives are used. Ensure validation studies for patient-specific cutting guides and implants.

**Table 6:** Best practice recommendations for PoC 3D printing centers.

Task	Considerations
Internal Regulation [180]	PoC centers should create a Quality Systems (QS) regulations document, detailing training, processes, parameters, and maintenance protocols. QS regulations should align with FDA standards under 21CFR820, even if Premarket Notification or Approval is not required.
FDA-Cleared Software	Use FDA-cleared and locally validated segmentation and CAD software for creating patient-specific diagnostic anatomic models. The software should meet FDA clearance for the intended indications.
Accurate Segmentation	Ensure that a radiologist and the ordering physician review and approve the segmentation for 3D-printed models. Document the training and qualifications of all segmentation personnel, including radiology technologists and industrial designers. For oncologic cases, a trained oncologist should segment tumor margins.
Validated Manufacturing Processes	Establish internal regulation processes, including installation qualification (IQ), operating qualification (OQ), and performance qualification (PQ). Document training for all personnel to ensure proper operation and up-to-date skills.
Biocompatibility Testing [178]	Devices used in sterile fields must undergo ISO 10993 [181] biocompatibility testing. Due to the complexity and cost, consider outsourcing these validation studies unless the institution has an accredited facility.
Legal Considerations	Engage with the institution’s legal team to discuss risks and liabilities associated with PoC 3D printing, especially when manufacturing devices previously purchased externally. This increases the hospital’s liability as the legal manufacturer if the implant is printed at the PoC.

## Data Availability

Data sharing is not applicable to this article.

## References

[1] Cheng B.C., Swink I., Yusufbekov R., Birgelen M., Ferrara L., Lewandrowski K.U., Coric D. (2020). Current Concepts of Contemporary Expandable Lumbar Interbody Fusion Cage Designs, Part 1: An Editorial on Their Biomechanical Characteristics. Int. J. Spine Surg..

[2] Godzik J., Lehrman J.N., Newcomb A., Menon R.K., Whiting A.C., Kelly B.P., Snyder L.A. (2019). Tailoring selection of transforaminal interbody spacers based on biomechanical characteristics and surgical goals: Evaluation of an expandable spacer. J. Neurosurg. Spine.

[3] Coe J.D., Zucherman J.F., Kucharzyk D.W., Poelstra K.A., Miller L.E., Kunwar S. (2016). Multiexpandable cage for minimally invasive posterior lumbar interbody fusion. Med. Devices.

[4] Cannestra A.F., Peterson M.D., Parker S.R., Roush T.F., Bundy J.V., Turner A.W. (2016). MIS Expandable Interbody Spacers: A Literature Review and Biomechanical Comparison of an Expandable MIS TLIF With Conventional TLIF and ALIF. Spine.

[5] Boktor J.G., Pockett R.D., Verghese N. (2018). The expandable transforaminal lumbar interbody fusion—Two years follow-up. J. Craniovertebr. Junction Spine.

[6] Barrett-Tuck R., Del Monaco D., Block J.E. (2017). One and two level posterior lumbar interbody fusion (PLIF) using an expandable, stand-alone, interbody fusion device: A VariLift((R)) case series. J. Spine Surg..

[7] Alvi M.A., Kurian S.J., Wahood W., Goyal A., Elder B.D., Bydon M. (2019). Assessing the Difference in Clinical and Radiologic Outcomes Between Expandable Cage and Nonexpandable Cage Among Patients Undergoing Minimally Invasive Transforaminal Interbody Fusion: A Systematic Review and Meta-Analysis. World Neurosurg..

[8] Alimi M., Shin B., Macielak M., Hofstetter C.P., Njoku I., Tsiouris A.J., Elowitz E., Hartl R. (2015). Expandable Polyaryl-Ether-Ether-Ketone Spacers for Interbody Distraction in the Lumbar Spine. Global Spine J..

[9] Lewandrowski K.U., Yeung A., Lorio M.P., Yang H., Ramírez León J.F., Sánchez J.A.S., Fiorelli R.K.A., Lim K.T., Moyano J., Dowling Á. (2023). Personalized Interventional Surgery of the Lumbar Spine: A Perspective on Minimally Invasive and Neuroendoscopic Decompression for Spinal Stenosis. J. Pers. Med..

[10] Lewandrowski K.U., Abraham I., Ramírez León J.F., Telfeian A.E., Lorio M.P., Hellinger S., Knight M., De Carvalho P.S.T., Ramos M.R.F., Dowling Á. (2022). A Proposed Personalized Spine Care Protocol (SpineScreen) to Treat Visualized Pain Generators: An Illustrative Study Comparing Clinical Outcomes and Postoperative Reoperations between Targeted Endoscopic Lumbar Decompression Surgery, Minimally Invasive TLIF and Open Laminectomy. J. Pers. Med..

[11] Ham D.W., Jung C.W., Chang D.G., Yang J.J., Song K.S. (2023). Feasibility of Non-window Three-Dimensional-Printed Porous Titanium Cage in Posterior Lumbar Interbody Fusion: A Pilot Trial. Clin. Orthop. Surg..

[12] Deng Z., Zou Q., Wang L., Wang L., Xiu P., Feng G., Song Y., Yang X. (2023). Comparison between Three-Dimensional Printed Titanium and PEEK Cages for Cervical and Lumbar Interbody Fusion: A Prospective Controlled Trial. Orthop. Surg..

[13] Duan Y., Feng D., Li T., Wang Y., Jiang L., Huang Y. (2024). Comparison of Lumbar Interbody Fusion with 3D-Printed Porous Titanium Cage Versus Polyetheretherketone Cage in Treating Lumbar Degenerative Disease: A Systematic Review and Meta-Analysis. World Neurosurg..

[14] Hahn B.-D., Park D.-S., Choi J.-J., Ryu J., Yoon W.-H., Choi J.-H., Kim J.-W., Ahn C.-W., Kim H.-E., Yoon B.-H. (2013). Osteoconductive hydroxyapatite coated PEEK for spinal fusion surgery. Appl. Surf. Sci..

[15] Gunzburg R., Colloca C.J., Jones C.F., Hall D.J., McAviney J., Callary S., Hegazy M.A., Szpalski M., Freeman B.J.C. (2019). Does nanoscale porous titanium coating increase lumbar spinal stiffness of an interbody fusion cage? An in vivo biomechanical analysis in an ovine model. Clin. Biomech..

[16] Torstrick F.B., Safranski D.L., Burkus J.K., Chappuis J.L., Lee C.S.D., Guldberg R.E., Gall K., Smith K.E. (2017). Getting PEEK to Stick to Bone: The Development of Porous PEEK for Interbody Fusion Devices. Tech. Orthop..

[17] Dufils J., Faverjon F., Héau C., Donnet C., Benayoun S., Valette S. (2017). Evaluation of a variety of aC: H coatings on PEEK for biomedical implants. Surf. Coat. Technol..

[18] Patel K., Doyle C.S., Yonekura D., James B.J. (2010). Effect of surface roughness parameters on thermally sprayed PEEK coatings. Surf. Coat. Technol..

[19] Torstrick F.B., Klosterhoff B.S., Westerlund L.E., Foley K.T., Gochuico J., Lee C.S., Gall K., Safranski D.L. (2018). Impaction durability of porous polyether-ether-ketone (PEEK) and titanium-coated PEEK interbody fusion devices. Spine J..

[20] Yao N., Wang W., Liu Y. (2011). Percutaneous endoscopic lumbar discectomy and interbody fusion with B-Twin expandable spinal spacer. Arch. Orthop. Trauma. Surg..

[21] Ramirez Leon J.F., Ardila A.S., Rugeles Ortiz J.G., Martinez C.R., Alonso Cuellar G.O., Infante J., Lewandrowski K.U. (2020). Standalone lordotic endoscopic wedge lumbar interbody fusion (LEW-LIF) with a threaded cylindrical peek cage: Report of two cases. J. Spine Surg..

[22] Neely W.F., Fichtel F., Del Monaco D.C., Block J.E. (2016). Treatment of Symptomatic Lumbar Disc Degeneration with the VariLift-L Interbody Fusion System: Retrospective Review of 470 Cases. Int. J. Spine Surg..

[23] Kale A., Oz I.I., Onk A., Kalayci M., Buyukuysal C. (2017). Unilaterally posterior lumbar interbody fusion with double expandable peek cages without pedicle screw support for lumbar disc herniation. Neurol. Neurochir. Pol..

[24] Emstad E., Del Monaco D.C., Fielding L.C., Block J.E. (2015). The VariLift((R)) Interbody Fusion System: Expandable, standalone interbody fusion. Med. Devices.

[25] Park J.H., Bae C.W., Jeon S.R., Rhim S.C., Kim C.J., Roh S.W. (2010). Clinical and radiological outcomes of unilateral facetectomy and interbody fusion using expandable cages for lumbosacral foraminal stenosis. J. Korean Neurosurg. Soc..

[26] Lewandrowski K.U. (2018). Surgical Technique of Endoscopic Transforaminal Decompression and Fusion with a Threaded Expandable Interbody Fusion Cage and A Report of 24 Cases. J. Spine.

[27] Coric D. (2019). Transforaminal/Posterior Lumbar Interbody Fusion With the FlareHawk^®^ Expandable Interbody Fusion Device: A Retrospective Chart Review Study.

[28] Zhang J., Pan A., Zhou L., Yu J., Zhang X. (2018). Comparison of unilateral pedicle screw fixation and interbody fusion with PEEK cage vs. standalone expandable fusion cage for the treatment of unilateral lumbar disc herniation. Arch. Med. Sci..

[29] Tassemeier T., Haversath M., Jager M. (2018). Transforaminal lumbar interbody fusion with expandable cages: Radiological and clinical results of banana-shaped and straight implants. J. Craniovertebr. Junction Spine.

[30] Morgenstern R., Morgenstern C. (2018). Feasibility of Full Percutaneous Segmental Stabilization of the Lumbar Spine With a Combination of an Expandable Interbody Cage and an Interspinous Spacer: Preliminary Results. Int. J. Spine Surg..

[31] Massie L.W., Zakaria H.M., Schultz L.R., Basheer A., Buraimoh M.A., Chang V. (2018). Assessment of radiographic and clinical outcomes of an articulating expandable interbody cage in minimally invasive transforaminal lumbar interbody fusion for spondylolisthesis. Neurosurg. Focus..

[32] Lewandrowski K.U., Ransom N.A., Yeung A. (2020). Subsidence induced recurrent radiculopathy after staged two-level standalone endoscopic lumbar interbody fusion with a threaded cylindrical cage: A case report. J. Spine Surg..

[33] Yildiz F., Yetim A., Alsaran A., Efeoglu I. (2009). Wear and corrosion behaviour of various surface treated medical grade titanium alloy in bio-simulated environment. Wear.

[34] Fan D., Li Y., Wang X., Zhu T., Wang Q., Cai H., Li W., Tian Y., Liu Z. (2020). Progressive 3D printing technology and its application in medical materials. Front. Pharmacol..

[35] Nadagouda M.N., Rastogi V., Ginn M. (2020). A review on 3D printing techniques for medical applications. Curr. Opin. Chem. Eng..

[36] Yan Q., Dong H., Su J., Han J., Song B., Wei Q., Shi Y. (2018). A review of 3D printing technology for medical applications. Engineering.

[37] MacDonald E., Wicker R. (2016). Multiprocess 3D printing for increasing component functionality. Science.

[38] Jing Z., Zhang T., Xiu P., Cai H., Wei Q., Fan D., Lin X., Song C., Liu Z. (2020). Functionalization of 3D-printed titanium alloy orthopedic implants: A literature review. Biomed. Mater..

[39] Fan H., Fu J., Li X., Pei Y., Li X., Pei G., Guo Z. (2015). Implantation of customized 3-D printed titanium prosthesis in limb salvage surgery: A case series and review of the literature. World J. Surg. Oncol..

[40] Song P., Hu C., Pei X., Sun J., Sun H., Wu L., Jiang Q., Fan H., Yang B., Zhou C. (2019). Dual modulation of crystallinity and macro-/microstructures of 3D printed porous titanium implants to enhance stability and osseointegration. J. Mater. Chem. B.

[41] Wallace N., Schaffer N.E., Aleem I.S., Patel R. (2020). 3D-printed Patient-specific Spine Implants: A Systematic Review. Clin. Spine Surg..

[42] Kaur M., Singh K. (2019). Review on titanium and titanium based alloys as biomaterials for orthopaedic applications. Mater. Sci. Eng. C.

[43] Khorasani A.M., Goldberg M., Doeven E.H., Littlefair G. (2015). Titanium in biomedical applications—properties and fabrication: A review. J. Biomater. Tissue Eng..

[44] Geetha M., Singh A.K., Asokamani R., Gogia A.K. (2009). Ti based biomaterials, the ultimate choice for orthopaedic implants–a review. Prog. Mater. Sci..

[45] Van Noort R. (1987). Titanium: The implant material of today. J. Mater. Sci..

[46] Amalraju D., Dawood A. (2012). Mechanical strength evaluation analysis of stainless steel and titanium locking plate for femur bone fracture. Eng. Sci. Technol. Int. J..

[47] Kyriakides T.R., Raj A., Tseng T.H., Xiao H., Nguyen R., Mohammed F.S., Halder S., Xu M., Wu M.J., Bao S. (2021). Biocompatibility of nanomaterials and their immunological properties. Biomed. Mater..

[48] He S., Zhu J., Jing Y., Long S., Tang L., Cheng L., Shi Z. (2024). Effect of 3D-Printed Porous Titanium Alloy Pore Structure on Bone Regeneration: A Review. Coatings.

[49] Kelly C.N., Wang T., Crowley J., Wills D., Pelletier M.H., Westrick E.R., Adams S.B., Gall K., Walsh W.R. (2021). High-strength, porous additively manufactured implants with optimized mechanical osseointegration. Biomaterials.

[50] El-Hajje A., Kolos E.C., Wang J.K., Maleksaeedi S., He Z., Wiria F.E., Choong C., Ruys A.J. (2014). Physical and mechanical characterisation of 3D-printed porous titanium for biomedical applications. J. Mater. Sci. Mater. Med..

[51] Murr L.E., Gaytan S., Medina F., Lopez H., Martinez E., Machado B., Hernandez D., Martinez L., Lopez M., Wicker R. (2010). Next-generation biomedical implants using additive manufacturing of complex, cellular and functional mesh arrays. Philos. Trans. R. Soc. A Math. Phys. Eng. Sci..

[52] Pałka K., Pokrowiecki R. (2018). Porous titanium implants: A review. Adv. Eng. Mater..

[53] Wang X., Xu S., Zhou S., Xu W., Leary M., Choong P., Qian M., Brandt M., Xie Y.M. (2016). Topological design and additive manufacturing of porous metals for bone scaffolds and orthopaedic implants: A review. Biomaterials.

[54] Frost B.A., Camarero-Espinosa S., Foster E.J. (2019). Materials for the spine: Anatomy, problems, and solutions. Materials.

[55] Wu Y., Liu J., Kang L., Tian J., Zhang X., Hu J., Huang Y., Liu F., Wang H., Wu Z. (2023). An overview of 3D printed metal implants in orthopedic applications: Present and future perspectives. Heliyon.

[56] Lee J.-A., Koh Y.-G., Kang K.-T. (2020). Biomechanical and clinical effect of patient-specific or customized knee implants: A review. J. Clin. Med..

[57] Wang Z., Mithieux S.M., Weiss A.S. (2019). Fabrication techniques for vascular and vascularized tissue engineering. Adv. Healthc. Mater..

[58] Holmes B., Bulusu K., Plesniak M., Zhang L.G. (2016). A synergistic approach to the design, fabrication and evaluation of 3D printed micro and nano featured scaffolds for vascularized bone tissue repair. Nanotechnology.

[59] Negendahl K. (2015). Building performance simulation in the early design stage: An introduction to integrated dynamic models. Autom. Constr..

[60] Chua C.K., Wong C.H., Yeong W.Y. (2017). Standards, Quality Control, and Measurement Sciences in 3D Printing and Additive Manufacturinged.

[61] Mitra I., Bose S., Dernell W.S., Dasgupta N., Eckstrand C., Herrick J., Yaszemski M.J., Goodman S.B., Bandyopadhyay A. (2021). 3D Printing in alloy design to improve biocompatibility in metallic implants. Mater. Today.

[62] Al-Tamimi A.A., Almeida H., Bartolo P. (2020). Structural optimisation for medical implants through additive manufacturing. Prog. Addit. Manuf..

[63] He F., Liu Q., Deng P. (2020). Investigation of the anisotropic characteristics of layered rocks under uniaxial compression based on the 3D printing technology and the combined finite-discrete element method. Adv. Mater. Sci. Eng..

[64] Morrison R.J., Kashlan K.N., Flanangan C.L., Wright J.K., Green G.E., Hollister S.J., Weatherwax K.J. (2015). Regulatory considerations in the design and manufacturing of implanTable 3D-printed medical devices. Clin. Transl. Sci..

[65] Martinez-Marquez D., Jokymaityte M., Mirnajafizadeh A., Carty C.P., Lloyd D., Stewart R.A. (2019). Development of 18 quality control gates for additive manufacturing of error free patient-specific implants. Materials.

[66] Gebhardt A., Hötter J.-S. (2016). Additive Manufacturing: 3D Printing for Prototyping and Manufacturinged.

[67] Verner I., Merksamer A. (2015). Digital design and 3D printing in technology teacher education. Procedia Cirp.

[68] Panesar A., Brackett D., Ashcroft I., Wildman R., Hague R. (2014). Design Optimization Strategy for Multifunctional 3D Printing.

[69] Mhmood T.R., Al-Karkhi N.K. (2023). A Review of the Stereo lithography 3D Printing Process and the Effect of Parameters on Quality. Al-Khwarizmi Eng. J..

[70] Aboulkhair N.T., Simonelli M., Parry L., Ashcroft I., Tuck C., Hague R. (2019). 3D printing of Aluminium alloys: Additive Manufacturing of Aluminium alloys using selective laser melting. Prog. Mater. Sci..

[71] Osipov V., Platonov V., Trigub M., Tikhonov E., Vasnev N., Gembukh P., Zubarev N., Kochurin E. (2024). Experimental study of melt splashing during yttrium oxide evaporation using ytterbium fiber laser. Int. J. Heat Mass Transf..

[72] Yap C.Y., Chua C.K., Dong Z.L., Liu Z.H., Zhang D.Q., Loh L.E., Sing S.L. (2015). Review of selective laser melting: Materials and applications. Appl. Phys. Rev..

[73] Yasa E. (2021). Selective laser melting: Principles and surface quality. Handbooks in Advanced Manufacturing.

[74] Koike M., Greer P., Owen K., Lilly G., Murr L.E., Gaytan S.M., Martinez E., Okabe T. (2011). Evaluation of titanium alloys fabricated using rapid prototyping technologies—electron beam melting and laser beam melting. Materials.

[75] Zhang L.C., Liu Y., Li S., Hao Y. (2018). Additive manufacturing of titanium alloys by electron beam melting: A review. Adv. Eng. Mater..

[76] Angjellari M., Tamburri E., Montaina L., Natali M., Passeri D., Rossi M., Terranova M.L. (2017). Beyond the concepts of nanocomposite and 3D printing: PVA and nanodiamonds for layer-by-layer additive manufacturing. Mater. Des..

[77] Azgomi N., Tetteh F., Duntu S.H., Boakye-Yiadom S. (2021). Effect of Heat Treatment on the Microstructural Evolution and Properties of 3D-Printed and Conventionally Produced Medical-Grade Ti6Al4V ELI Alloy. Metall. Mater. Trans. A.

[78] Diniță A., Neacșa A., Portoacă A.I., Tănase M., Ilinca C.N., Ramadan I.N. (2023). Additive manufacturing post-processing treatments, a review with emphasis on mechanical characteristics. Materials.

[79] Bernhardt A., Schneider J., Schroeder A., Papadopoulous K., Lopez E., Brückner F., Botzenhart U. (2021). Surface conditioning of additively manufactured titanium implants and its influence on materials properties and in vitro biocompatibility. Mater. Sci. Eng. C.

[80] Wiseman J., Rawther T., Langbart M., Kernohan M., Ngo Q. (2022). Sterilization of bedside 3D-printed devices for use in the operating room. Ann. 3D Print. Med..

[81] Sahoo P., Das S.K. (2019). Emerging trends in additive and subtractive manufacturing. Addit. Subtractive Manuf..

[82] Aliakbari M. (2012). Additive Manufacturing: State-of-the-Art, Capabilities, and Sample Applications with Cost Analysis. Master’s Thesis.

[83] Fogel G., Martin N., Lynch K., Pelletier M.H., Wills D., Wang T., Walsh W.R., Williams G.M., Malik J., Peng Y. (2022). Subsidence and fusion performance of a 3D-printed porous interbody cage with stress-optimized body lattice and microporous endplates-a comprehensive mechanical and biological analysis. Spine J..

[84] Kang H., Hollister S.J., La Marca F., Park P., Lin C.-Y. (2013). Porous biodegradable lumbar interbody fusion cage design and fabrication using integrated global-local topology optimization with laser sintering. J. Biomech. Eng..

[85] Albrektsson T., Johansson C. (2001). Osteoinduction, osteoconduction and osseointegration. Eur. Spine J..

[86] Borden M., Attawia M., Laurencin C.T. (2002). The sintered microsphere matrix for bone tissue engineering: In vitro osteoconductivity studies. J. Biomed. Mater. Res..

[87] de Wild M., Schumacher R., Mayer K., Schkommodau E., Thoma D., Bredell M., Kruse Gujer A., Grätz K.W., Weber F.E. (2013). Bone regeneration by the osteoconductivity of porous titanium implants manufactured by selective laser melting: A histological and micro computed tomography study in the rabbit. Tissue Eng. Part A.

[88] Liu Y., Zhang R., Liu S., Sun J., Zhang X., Kang P., Zhang R., Yang Y., Li R. (2021). The Variability in Cytocompatibility and Bone Conduction Based on Different Pore Size and Porosity of N-HA/PA66 Composite Scaffolds. Front. Mater..

[89] de Wild M., Zimmermann S., Rüegg J., Schumacher R., Fleischmann T., Ghayor C., Weber F.E. (2016). Influence of microarchitecture on osteoconduction and mechanics of porous titanium scaffolds generated by selective laser melting. 3D Print. Addit. Manuf..

[90] Bai Y., Wang Z., He X., Zhu Y., Xu X., Yang H., Mei G., Chen S., Ma B., Zhu R. (2024). Application of Bioactive Materials for Osteogenic Function in Bone Tissue Engineering. Small Methods.

[91] Andrade J.V. (1986). Hlady in Protein Adsorption and Materials Biocompatibility: A Tutorial Review and Suggested Hypotheses.

[92] Wilson C.J., Clegg R.E., Leavesley D.I., Pearcy M.J. (2005). Mediation of biomaterial–cell interactions by adsorbed proteins: A review. Tissue Eng..

[93] Jansson E., Tengvall P. (2001). In vitro preparation and ellipsometric characterization of thin blood plasma clot films on silicon. Biomaterials.

[94] Keselowsky B.G., Collard D.M., García A.J. (2003). Surface chemistry modulates fibronectin conformation and directs integrin binding and specificity to control cell adhesion. J. Biomed. Mater. Res..

[95] Gittens R.A., Olivares-Navarrete R., Schwartz Z., Boyan B.D. (2014). Implant osseointegration and the role of microroughness and nanostructures: Lessons for spine implants. Acta Biomater..

[96] Marx R.E. (2004). Platelet-rich plasma: Evidence to support its use. J. Oral Maxillofac. Surg..

[97] Babensee J.E., Anderson J.M., McIntire L.V., Mikos A.G. (1998). Host response to tissue engineered devices. Adv. Drug Deliv. Rev..

[98] Schindeler A., McDonald M.M., Bokko P., Little D.G. (2008). Bone remodeling during fracture repair: The cellular picture. Seminars in Cell & Developmental Biology.

[99] Bruder S.P., Fink D.J., Caplan A.I. (1994). Mesenchymal stem cells in bone development, bone repair, and skeletal regenaration therapy. J. Cell. Biochem..

[100] Olivares-Navarrete R., Gittens R.A., Schneider J.M., Hyzy S.L., Haithcock D.A., Ullrich P.F., Schwartz Z., Boyan B.D. (2012). Osteoblasts exhibit a more differentiated phenotype and increased bone morphogenetic protein production on titanium alloy substrates than on poly-ether-ether-ketone. Spine J..

[101] Davies J. (1998). Mechanisms of endosseous integration. Int. J. Prosthodont..

[102] Saruwatari L., Aita H., Butz F., Nakamura H.K., Ouyang J., Yang Y., Chiou W.A., Ogawa T. (2005). Osteoblasts generate harder, stiffer, and more delamination-resistant mineralized tissue on titanium than on polystyrene, associated with distinct tissue micro-and ultrastructure. J. Bone Miner. Res..

[103] Owen T.A., Aronow M., Shalhoub V., Barone L.M., Wilming L., Tassinari M.S., Kennedy M.B., Pockwinse S., Lian J.B., Stein G.S. (1990). Progressive development of the rat osteoblast phenotype in vitro: Reciprocal relationships in expression of genes associated with osteoblast proliferation and differentiation during formation of the bone extracellular matrix. J. Cell. Physiol..

[104] Mulari M., Qu Q., Härkönen P., Väänänen H. (2004). Osteoblast-like cells complete osteoclastic bone resorption and form new mineralized bone matrix in vitro. Calcif. Tissue Int..

[105] Piattelli A., Scarano A., Favero L., Iezzi G., Petrone G., Favero G.A. (2003). Clinical and histologic aspects of dental implants removed due to mobility. J. Periodontol..

[106] McGilvray K.C., Easley J., Seim H.B., Regan D., Berven S.H., Hsu W.K., Mroz T.E., Puttlitz C.M. (2018). Bony ingrowth potential of 3D-printed porous titanium alloy: A direct comparison of interbody cage materials in an in vivo ovine lumbar fusion model. Spine J..

[107] Laratta J.L., Vivace B.J., López-Peña M., Guzón F.M., Gonzalez-Cantalpeidra A., Jorge-Mora A., Villar-Liste R.M., Pino-Lopez L., Lukyanchuk A., Taghizadeh E.A. (2022). 3D-printed titanium cages without bone graft outperform PEEK cages with autograft in an animal model. Spine J..

[108] Prolo D.J., Rodrigo J.J. (1985). Contemporary bone graft physiology and surgery. Clin. Orthop. Relat. Res..

[109] Malone H., Mundis G.M., Collier M., Kidwell R.L., Rios F., Jelousi M., Galli S., Shahidi B., Akbarnia B.A., Eastlack R.K. (2022). Can a bioactive interbody device reduce the cost burden of achieving lateral lumbar fusion?. J. Neurosurg. Spine.

[110] Chang S.Y., Kang D.-H., Cho S.K. (2023). Innovative Developments in Lumbar Interbody Cage Materials and Design: A Comprehensive Narrative Review. Asian Spine J..

[111] Castilho M., Dias M., Vorndran E., Gbureck U., Fernandes P., Pires I., Gouveia B., Armés H., Pires E., Rodrigues J. (2014). Application of a 3D printed customized implant for canine cruciate ligament treatment by tibial tuberosity advancement. Biofabrication.

[112] Wong K.C. (2016). 3D-printed patient-specific applications in orthopedics. Orthop. Res. Rev..

[113] Sun T., He X., Li Z. (2023). Digital twin in healthcare: Recent updates and challenges. Digit. Health.

[114] Lu Y. (2017). Cyber physical system (CPS)-based industry 4.0: A survey. J. Ind. Integr. Manag..

[115] Attia P.M., Grover A., Jin N., Severson K.A., Markov T.M., Liao Y.-H., Chen M.H., Cheong B., Perkins N., Yang Z. (2020). Closed-loop optimization of fast-charging protocols for batteries with machine learning. Nature.

[116] Vallée A. (2023). Digital twin for healthcare systems. Front. Digit. Health.

[117] Gámez Díaz R., Yu Q., Ding Y., Laamarti F., El Saddik A. (2020). Digital twin coaching for physical activities: A survey. Sensors.

[118] Anderson A.M. (2021). Towards Digital Twinning for Additive Manufacturing of Medical Implants. Master’s Thesis.

[119] Thayaparan G.K., Owbridge M.G., Linden M., Thompson R.G., Lewis P.M., D’Urso P.S. (2020). Measuring the performance of patient-specific solutions for minimally invasive transforaminal lumbar interbody fusion surgery. J. Clin. Neurosci..

[120] Parr W.C., Burnard J.L., Wilson P.J., Mobbs R.J. (2019). 3D printed anatomical (bio) models in spine surgery: Clinical benefits and value to health care providers. J. Spine Surg..

[121] Sun X., Yang E., Zhao C., Cheng X., Zhang K., Tian H., Ding B., Li H., Jiang W., Dai K. (2021). Progress in the application of 3D printing technology in spine surgery. J. Shanghai Jiaotong Univ..

[122] Mobbs R.J., Parr W.C., Choy W.J., McEvoy A., Walsh W.R., Phan K. (2019). Anterior lumbar interbody fusion using a personalized approach: Is custom the future of implants for anterior lumbar interbody fusion surgery?. World Neurosurg..

[123] Ling Q., He E., Ouyang H., Guo J., Yin Z., Huang W. (2018). Design of mulitlevel OLF approach (“V”-shaped decompressive laminoplasty) based on 3D printing technology. Eur. Spine J..

[124] Fiani B., Newhouse A., Cathel A., Sarhadi K., Soula M. (2021). Implications of 3-Dimensional Printed Spinal Implants on the Outcomes in Spine Surgery. J. Korean Neurosurg. Soc..

[125] Gerling M.C., Hale S.D., White-Dzuro C., Pierce K.E., Naessig S.A., Ahmad W., Passias P.G. (2019). Ambulatory spine surgery. J. Spine Surg..

[126] Xiao J., Huang W., Yang X., Yan W., Song D., Wei H., Liu T., Wu Z., Yang C. (2016). En bloc resection of primary malignant bone tumor in the cervical spine based on 3-dimensional printing technology. Orthop. Surg..

[127] Ahmed A.K., Pennington Z., Molina C.A., Xia Y., Goodwin C.R., Sciubba D.M. (2019). Multidisciplinary surgical planning for en bloc resection of malignant primary cervical spine tumors involving 3D-printed models and neoadjuvant therapies: Report of 2 cases. J. Neurosurg. Spine.

[128] Wei R., Guo W., Ji T., Zhang Y., Liang H. (2017). One-step reconstruction with a 3D-printed, custom-made prosthesis after total en bloc sacrectomy: A technical note. Eur. Spine J..

[129] Xu N., Wei F., Liu X., Jiang L., Cai H., Li Z., Yu M., Wu F., Liu Z. (2016). Reconstruction of the upper cervical spine using a personalized 3D-printed vertebral body in an adolescent with Ewing sarcoma. Spine.

[130] Li X., Wang Y., Zhao Y., Liu J., Xiao S., Mao K. (2017). Multilevel 3D printing implant for reconstructing cervical spine with metastatic papillary thyroid carcinoma. Spine.

[131] Kim D., Lim J.-Y., Shim K.-W., Han J.W., Yi S., Yoon D.H., Kim K.N., Ha Y., Ji G.Y., Shin D.A. (2017). Sacral reconstruction with a 3D-printed implant after hemisacrectomy in a patient with sacral osteosarcoma: 1-year follow-up result. Yonsei Med. J..

[132] Choy W.J., Mobbs R.J., Wilcox B., Phan S., Phan K., Sutterlin C.E. (2017). Reconstruction of thoracic spine using a personalized 3D-printed vertebral body in adolescent with T9 primary bone tumor. World Neurosurg..

[133] Mobbs R.J., Coughlan M., Thompson R., Sutterlin C.E., Phan K. (2017). The utility of 3D printing for surgical planning and patient-specific implant design for complex spinal pathologies: Case report. J. Neurosurg. Spine.

[134] Chin B.Z., Ji T., Tang X., Yang R., Guo W. (2019). Three-Level Lumbar En Bloc Spondylectomy with Three-Dimensional-Printed Vertebrae Reconstruction for Recurrent Giant Cell Tumor. World Neurosurg..

[135] Yang M., Li C., Li Y., Zhao Y., Wei X., Zhang G., Fan J., Ni H., Chen Z., Bai Y. (2015). Application of 3D rapid prototyping technology in posterior corrective surgery for Lenke 1 adolescent idiopathic scoliosis patients. Medicine.

[136] Tu Q., Ding H.W., Chen H., Miao Q.J., Yang X., Li K., Zhang K., Wu Z.H., Tang Y., Xia H. (2019). Three-Dimensional-Printed Individualized Guiding Templates for Surgical Correction of Severe Kyphoscoliosis Secondary to Ankylosing Spondylitis: Outcomes of 9 Cases. World Neurosurg..

[137] Rosenzweig D.H., Carelli E., Steffen T., Jarzem P., Haglund L. (2015). 3D-printed ABS and PLA scaffolds for cartilage and nucleus pulposus tissue regeneration. Int. J. Mol. Sci..

[138] Lu T., Liu C., Yang B., Liu J., Zhang F., Wang D., Li H., He X. (2017). Single-level anterior cervical corpectomy and fusion using a new 3D-printed anatomy-adaptive titanium mesh cage for treatment of cervical spondylotic myelopathy and ossification of the posterior longitudinal ligament: A retrospective case series study. Med. Sci. Monit. Int. Med. J. Exp. Clin. Res..

[139] Siu T.L., Rogers J.M., Lin K., Thompson R., Owbridge M. (2018). Custom-made titanium 3-dimensional printed interbody cages for treatment of osteoporotic fracture–related spinal deformity. World Neurosurg..

[140] Thayaparan G.K., Owbridge M.G., Thompson R.G., D’Urso P.S. (2018). Designing patient-specific 3D printed devices for posterior atlantoaxial transarticular fixation surgery. J. Clin. Neurosci..

[141] Choy W.J., Parr W.C., Phan K., Walsh W.R., Mobbs R.J. (2018). 3-dimensional printing for anterior cervical surgery: A review. J. Spine Surg..

[142] Mokawem M., Katzouraki G., Harman C.L., Lee R. (2019). Lumbar interbody fusion rates with 3D-printed lamellar titanium cages using a silicate-substituted calcium phosphate bone graft. J. Clin. Neurosci..

[143] Toop N., Dhaliwal J., Grossbach A., Gibbs D., Reddy N., Keister A., Mallory N., Xu D., Viljoen S. (2003). Subsidence Rates Associated With Porous 3D-Printed Versus Solid Titanium Cages in Transforaminal Lumbar Interbody Fusion. Glob. Spine J..

[144] Alan N., Vodovotz L., Muthiah N., Deng H., Guha D., Agarwal N., Ozpinar A., Mushlin H.M., Puccio L., Hamilton D.K. (2022). Subsidence after lateral lumbar interbody fusion using a 3D-printed porous titanium interbody cage: Single-institution case series. J. Neurosurg. Spine.

[145] Adl Amini D., Okano I., Oezel L., Zhu J., Chiapparelli E., Shue J., Sama A.A., Cammisa F.P., Girardi F.P., Hughes A.P. (2021). Evaluation of cage subsidence in standalone lateral lumbar interbody fusion: Novel 3D-printed titanium versus polyetheretherketone (PEEK) cage. Eur. Spine J..

[146] Fernandes R.J.R. (2021). Biomechanical Strategies to Reduce Subsidence in Posterior Lumbar Interbody Fusion Procedures.

[147] Class 2 Device Recall Tritanium Posterior Lumbar (PL) Cage. [Access data.fda.gov.]. 13 December 2018. https://www.accessdata.fda.gov/scripts/cdrh/cfdocs/cfRES/res.cfm?id=169021.

[148] Tang H., Zhao P., Xiang C., Liu N., Jia L. (2018). Ti-6Al-4V orthopedic implants made by selective electron beam melting. Titanium in Medical and Dental Applications.

[149] Amelot A., Colman M., Loret J.-E. (2018). Vertebral body replacement using patient-specific three–dimensional-printed polymer implants in cervical spondylotic myelopathy: An encouraging preliminary report. Spine J..

[150] Chung S.-S., Lee K.-J., Kwon Y.-B., Kang K.-C. (2017). Characteristics and efficacy of a new 3-dimensional printed mesh structure titanium alloy spacer for posterior lumbar interbody fusion. Orthopedics.

[151] Thayaparan G.K., Owbridge M.G., Thompson R.G., D’Urso P.S. (2019). Designing patient-specific solutions using biomodelling and 3D-printing for revision lumbar spine surgery. Eur. Spine J..

[152] Wei R., Guo W., Yang R., Tang X., Yang Y., Ji T., Liang H. (2019). Reconstruction of the pelvic ring after total en bloc sacrectomy using a 3D-printed sacral endoprosthesis with re-establishment of spinopelvic stability: A retrospective comparative study. Bone Jt. J..

[153] (2020). Global burden of 369 diseases and injuries in 204 countries and territories, 1990–2019, a systematic analysis for the Global Burden of Disease Study 2019. Lancet.

[154] Girolami M., Boriani S., Bandiera S., Barbanti-Bródano G., Ghermandi R., Terzi S., Tedesco G., Evangelisti G., Pipola V., Gasbarrini A. (2018). Biomimetic 3D-printed custom-made prosthesis for anterior column reconstruction in the thoracolumbar spine: A tailored option following en bloc resection for spinal tumors: Preliminary results on a case-series of 13 patients. Eur. Spine J..

[155] Mobbs R.J., Choy W.J., Wilson P., McEvoy A., Phan K., Parr W.C. (2018). L5 en-bloc vertebrectomy with customized reconstructive implant: Comparison of patient-specific versus off-the-shelf implant. World Neurosurg..

[156] Chung K.S., Shin D.A., Kim K.N., Ha Y., Yi S. (2019). Vertebral reconstruction with customized 3-dimensional− printed spine implant replacing large vertebral defect with 3-year follow-up. World Neurosurg..

[157] Phan K., Sgro A., Maharaj M.M., D’Urso P., Mobbs R.J. (2016). Application of a 3D custom printed patient specific spinal implant for C1/2 arthrodesis. J. Spine Surg..

[158] Spetzger U., Frasca M., König S.A. (2016). Surgical planning, manufacturing and implantation of an individualized cervical fusion titanium cage using patient-specific data. Eur. Spine J..

[159] He S., Yang X., Yang J., Ye C., Liu W., Wei H., Xiao J. (2019). Customized “whole-cervical-vertebral-body” reconstruction after modified subtotal spondylectomy of C2–C7 spinal tumor via piezoelectric surgery. Oper. Neurosurg..

[160] Zhang Y.-W., Deng L., Zhang X.-X., Yu X.-L., Ai Z.-Z., Mei Y.-X., He F., Yu H., Zhang L., Xiao X. (2019). Three-dimensional printing-assisted cervical anterior bilateral pedicle screw fixation of artificial vertebral body for cervical tuberculosis. World Neurosurg..

[161] Rao P.J., Pelletier M.H., Walsh W.R., Mobbs R.J. (2014). Spine interbody implants: Material selection and modification, functionalization and bioactivation of surfaces to improve osseointegration. Orthop. Surg..

[162] Tan X., Tan Y., Chow C., Tor S., Yeong W. (2017). Metallic powder-bed based 3D printing of cellular scaffolds for orthopaedic implants: A state-of-the-art review on manufacturing, topological design, mechanical properties and biocompatibility. Mater. Sci. Eng. C.

[163] Pucci J.U., Christophe B.R., Sisti J.A., Connolly E.S. (2017). Three-dimensional printing: Technologies, applications, and limitations in neurosurgery. Biotechnol. Adv..

[164] (2024). Aetna’s Ruling Threatens Patient Choice and Clinical Excellence. [Vertebral Columns, Winter 2024]. https://isass.org/news-vertebralcolumns-winter-2024/.

[165] Smith J.S., Mundis G.M., Osorio J.A., Nicolau R.J., Temple-Wong M., Lafage R., Bess S., Ames C.P. (2023). Analysis of Personalized Interbody Implants in the Surgical Treatment of Adult Spinal Deformity. Glob. Spine J..

[166] Drossopoulos P.N., Ononogbu-Uche F.C., Tabarestani T.Q., Huang C.-C., Paturu M., Bardeesi A., Ray W.Z., Shaffrey C.I., Goodwin C.R., Erickson M. (2024). Evolution of the Transforaminal Lumbar Interbody Fusion (TLIF): From Open to Percutaneous to Patient-Specific. J. Clin. Med..

[167] 3D Printing in Drug Development & Emerging Health Care. https://www.fda.gov/media/125479/download.

[168] Beitler B.G., Abraham P.F., Glennon A.R., Tommasini S.M., Lattanza L.L., Morris J.M., Wiznia D.H. (2022). Interpretation of regulatory factors for 3D printing at hospitals and medical centers, or at the point of care. 3D Print. Med..

[169] Di Prima M., Coburn J., Hwang D., Kelly J., Khairuzzaman A., Ricles L. (2016). Additively manufactured medical products—the FDA perspective. 3D Print. Med..

[170] Technical Considerations for Additive Manufactured Medical Devices. https://www.fda.gov/media/97633/download.

[171] Arce K., Morris J., Alexander A., Ettinger K. (2020). Developing a Point-of-Care Manufacturing Program for Craniomaxillofacial Surgery. Atlas Oral. Maxillofac. Surg. Clin. N. Am..

[172] FDA/CDRH–RSNA SIG Joint Meeting on 3D Printed Patient-Specific Anatomic Models. https://wayback.archive-it.org/7993/20201222130156/https://www.fda.gov/medical-devices/workshops-conferences-medical-devices/fdacdrh-rsna-sig-joint-meeting-3d-printed-patient-specific-anatomic-models-august-31-2017.

[173] Mitsouras D., Liacouras P., Imanzadeh A., Giannopoulos A.A., Cai T., Kumamaru K.K., George E., Wake N., Caterson E.J., Pomahac B. (2015). Medical 3D Printing for the Radiologist. Radiographics.

[174] Mitsouras D., Liacouras P.C., Wake N., Rybicki F.J. (2020). RadioGraphics Update: Medical 3D Printing for the Radiologist. Radiographics.

[175] Christensen A., Rybicki F.J. (2017). Maintaining safety and efficacy for 3D printing in medicine. 3D Print. Med..

[176] Discussion Paper. 3D Printing Medical Devices at the Point of Care. https://www.fda.gov/medical-devices/3d-printing-medical-devices/3d-printing-medical-devices-point-care-discussion-paper.

[177] Content of Premarket Submissions for Management of Cybersecurity in Medical Devices. https://www.fda.gov/regulatory-information/search-fda-guidance-documents/content-premarket-submissions-management-cybersecurity-medical-devices-0.

[178] Chepelev L., Wake N., Ryan J., Althobaity W., Gupta A., Arribas E., Santiago L., Ballard D.H., Wang K.C., Weadock W. (2018). Radiological Society of North America (RSNA) 3D printing Special Interest Group (SIG): Guidelines for medical 3D printing and appropriateness for clinical scenarios. 3D Print. Med..

[179] Use of International Standard ISO 10993-1. “Biological Evaluation of Medical Devices—Part 1, Evaluation and Testing within a Risk Management Process. https://www.fda.gov/regulatory-information/search-fda-guidance-documents/use-international-standard-iso-10993-1-biological-evaluation-medical-devices-part-1-evaluation-and.

[180] Schulze M., Gosheger G., Bockholt S., De Vaal M., Budny T., Tönnemann M., Pützler J., Bövingloh A.S., Rischen R., Hofbauer V. (2021). Complex Bone Tumors of the Trunk-The Role of 3D Printing and Navigation in Tumor Orthopedics: A Case Series and Review of the Literature. J. Pers. Med..

[181] Thangaraju P., Varthya S.B. (2022). ISO 10993: Biological evaluation of medical devices. Medical Device Guidelines and Regulations Handbook.

[182] Mobarak M.H., Islam M.A., Hossain N., Al Mahmud M.Z., Rayhan M.T., Nishi N.J., Chowdhury M.A. (2023). Recent advances of additive manufacturing in implant fabrication—A review. Appl. Surf. Sci. Adv..

[183] Senkoylu A., Daldal I., Cetinkaya M. (2020). 3D printing and spine surgery. J. Orthop. Surg..

[184] Pei X., Wu L., Zhou C., Fan H., Gou M., Li Z., Zhang B., Lei H., Sun H., Liang J. (2020). 3D printed titanium scaffolds with homogeneous diamond-like structures mimicking that of the osteocyte microenvironment and its bone regeneration study. Biofabrication.

